# Transmission of Bacterial Symbionts With and Without Genome Erosion Between a Beetle Host and the Plant Environment

**DOI:** 10.3389/fmicb.2021.715601

**Published:** 2021-09-22

**Authors:** Jürgen C. Wierz, Paul Gaube, Dagmar Klebsch, Martin Kaltenpoth, Laura V. Flórez

**Affiliations:** ^1^Department of Evolutionary Ecology, Institute of Organismic and Molecular Evolution, Johannes Gutenberg University, Mainz, Germany; ^2^Molecular Biodiversity Research Group, Center for Computational and Theoretical Biology, Julius Maximilian University of Würzburg, Würzburg, Germany; ^3^Department of Insect Symbiosis, Max Planck Institute for Chemical Ecology, Jena, Germany; ^4^Department of Plant and Environmental Sciences, Section for Organismal Biology, University of Copenhagen, Copenhagen, Denmark

**Keywords:** insect symbiosis, mixed-mode transmission, *Burkholderia*, Lagriinae, environmental symbiont acquisition, Coleoptera, insect-plant-microbe, reduced genome

## Abstract

Many phytophagous insects harbor symbiotic bacteria that can be transmitted vertically from parents to offspring, or acquired horizontally from unrelated hosts or the environment. In the latter case, plants are a potential route for symbiont transfer and can thus foster a tripartite interaction between microbe, insect, and plant. Here, we focus on two bacterial symbionts of the darkling beetle *Lagria villosa* that belong to the genus *Burkholderia*; the culturable strain *B. gladioli* Lv-StA and the reduced-genome strain *Burkholderia* Lv-StB. The strains can be transmitted vertically and confer protection to the beetle’s eggs, but Lv-StA can also proliferate in plants, and both symbiont strains have presumably evolved from plant pathogens. Notably, little is known about the role of the environment for the transmission dynamics and the maintenance of the symbionts. Through manipulative assays, we demonstrate the transfer of the symbionts from the beetle to wheat, rice and soybean plants, as well as leaf litter. In addition, we confirm that aposymbiotic larvae can pick up Lv-StA from dry leaves and the symbiont can successfully establish in the beetle’s symbiotic organs. Also, we show that the presence of plants and soil in the environment improves symbiont maintenance. These results indicate that the symbionts of *L. villosa* beetles are still capable of interacting with plants despite signatures of genome erosion and suggest that a mixed-mode of bacterial transmission is likely key for the persistence of the symbiosis.

## Introduction

Due to their metabolic potential, microorganisms can be valuable symbiotic partners for plants and animals, opening new ecological possibilities for their hosts (Margulis and Fester, 1991). By providing specific nutrients, digestive enzymes, means for detoxification or protective compounds, microbial symbionts can facilitate living on demanding diets or surviving in otherwise challenging habitats. After initial establishment of a symbiosis, co-evolution can further enhance dependence (Fisher et al., 2017) and thus the interaction can become increasingly tight over time, occasionally progressing toward an obligate association (Aanen et al., 2009; Lamelas et al., 2011). Generally, the provision of key nutrients such as essential amino acids or vitamins lead to a higher reliance on symbiosis than context-dependent benefits, like defense against antagonists. Although dependence has been extensively studied in symbionts with a long history of association to their host, the factors driving the maintenance of symbiosis in more recent associations are less well understood.

The mechanism by which symbionts are transmitted is largely intertwined with the evolution of dependence. Hosts that are more dependent on their symbionts are more likely to transmit these vertically, i.e., from parent to offspring, as this ensures the benefit for the next generation (Bright and Bulgheresi, 2010; Fisher et al., 2017). Insects have evolved elaborate mechanisms to do so, including transovarial infection, egg smearing or delivery of symbionts in capsules, or jelly-like secretions (Douglas, 1989; Kikuchi, 2009; Salem et al., 2015). Nonetheless, many animals and plants acquire their symbionts *via* horizontal transmission from the environment (Kikuchi et al., 2007; Brune, 2016; Hartmann et al., 2017) or through mixed-mode transmission, a combination of both vertical and horizontal mechanisms (Ebert, 2013). Importantly, strict vertical transmission and increasing dependence are usually associated with severe genomic consequences for bacterial symbionts. In general, vertically transmitted symbionts have a smaller genome size than those that are horizontally transmitted (Fisher et al., 2017). Relaxed selection on genes that are not pivotal in a stable host environment, in combination with severe population bottlenecks during vertical transmission, leads to genome degradation over evolutionary time (Muller, 1964; Moran, 1996; McCutcheon et al., 2019). As a consequence, obligate symbiotic bacteria of several insects have tiny genomes with just a few hundred genes, often retaining only essential metabolic pathways that are important for the host (McCutcheon and Moran, 2011). The process of symbiont genome degradation frequently results in a loss of functions necessary for life outside the host and thus an irreversible evolutionary dead-end. However, occasional horizontal transmission can strongly influence the architecture of the symbiont genome and counteract this process (Brandvain et al., 2011) by affecting population size and gene flow (Russell et al., 2017).

In herbivorous insects, horizontal transmission of symbionts can be plant-mediated (Caspi-Fluger et al., 2012; Chrostek et al., 2017) and in some cases these bacteria can have negative effects on the plants (Paine et al., 1997). Insect-associated bacteria might even shift to a plant pathogenic lifestyle due to repeated exposure (Bressan et al., 2012). Other bacteria, which are adapted to an arthropod-associated and intracellular lifestyle, can be transmitted to plant hosts without causing any apparent disease, like *Rickettsia*, *Cardinium*, and *Wolbachia* (Caspi-Fluger et al., 2012; Gonella et al., 2015; Li et al., 2017). However, how these bacteria colonize and survive in the plant host, and whether the infection can be maintained on the long run is mostly unknown (Chrostek et al., 2017). Alternatively, the insect can become a primary host to a previously plant-associated bacterium (Nadarasah and Stavrinides, 2011), as was likely the case for the *Burkholderia* symbionts of Lagriinae beetles (Flórez et al., 2017).

The polyphagous herbivore *Lagria villosa* (Tenebrionidae: Lagriinae) lives in symbiosis with *Burkholderia* bacteria, which produce bioactive secondary metabolites and thereby inhibit the growth of pathogenic fungi on the beetle eggs (Flórez et al., 2017, 2018). The symbionts, which are hosted extracellularly in accessory glands of adult females and in specialized cuticular invaginations in the larvae, can be transmitted vertically (Stammer, 1929; Flórez et al., 2017) and presumably also horizontally (Flórez et al., 2017). Previous findings on different Lagriinae beetle species show that all symbiotic *Burkholderia* strains fall within the *B. gladioli* clade, and likely have plant-associated ancestors (Flórez et al., 2017). Although *B. gladioli* has a diverse host range (Compant et al., 2008; Jones et al., 2021) and closely related strains can have beneficial effects on plants (Balandreau and Mavingui, 2007; Shehata et al., 2016), it is mostly known as a phytopathogen (Gonzalez et al., 2007). Importantly, at least three different *B. gladioli* strains and various other bacteria can infect *L. villosa* (Flórez et al., 2018). The strain *Burkholderia* sp. Lv-StB (henceforth “Lv-StB”) is the most abundant and consistent bacterium in both the female accessory glands and eggs (Flórez et al., 2017, 2018), which is in line with a vertical transmission route. This strain is so far uncultured and presumed to play an important role in the defensive symbiosis. It carries a gene cluster for the biosynthesis of lagriamide (lga), a potentially key compound in the antifungal defense of the eggs (Flórez et al., 2018). Interestingly, the genome of Lv-StB is only 2.3Mbp in size, corresponding to about one fourth of the genome size of *B. gladioli* Lv-StA (henceforth “Lv-StA,” genome size 8.5Mbp), a closely related strain isolated from the same host species (Flórez et al., 2018; Waterworth et al., 2020). This difference is most likely due to genome erosion in Lv-StB as a result of the symbiotic relationship and suggests higher dependence to the insect host in comparison to Lv-StA, which can be readily cultivated *in vitro*.

Here, we investigate the transmission dynamics in the multipartite interaction between *L. villosa* beetles, mutualistic *Burkholderia* symbionts, and their environment. Considering the putative evolutionary background of the symbiont as plant-associated bacteria, we evaluate whether the symbionts can be transmitted to and from plants. Also, since previous observations indicate that symbiotic *Burkholderia* are lost in laboratory conditions (Flórez and Kaltenpoth, 2017), we evaluated the impact of the plant environment in the stability of the symbiosis with *Burkholderia*. These experiments demonstrate the transmission of the reduced-genome bacterial symbiont Lv-StB to plants. The bacterium can be transferred to various food plant species and to leaf litter from the adult host and persist in these environments for at least several days. Furthermore, the closely related Lv-StA can be picked up by the host larva from leaf litter and establishes in the adult symbiotic organs. Notably, when maintained in a semi-natural environment with plants and soil, there is a higher chance that *L. villosa* beetles maintain their *Burkholderia* symbionts. These findings indicate a mixed-mode transmission involving both loosely and tightly associated symbiont strains of *L. villosa* beetles.

## Materials and Methods

### Beetle Collection and Standard Rearing

*Lagria villosa* beetles (Supplementary Figure 1A) were collected from soybean, manioc, corn, and coffee plantations between January and February 2015 as well as in April 2018 in the state of São Paulo, Brazil. The beetles were kept in ventilated plastic containers under laboratory conditions at 23–26°C, 60% humidity, and a 16L:8D photoperiod unless indicated differently for specific experiments. They were supplied with soybean leaves, iceberg lettuce, and cotton soaked with autoclaved tap water *ad libitum*.

### Standard Sowing for Semi Sterile Plant Rearing

Sterile flower pots (Ø 6cm, 5cm height) were filled to about 75% of their volume with autoclaved vermiculite. Plant seeds were cleaned in 2% NaClO for 2min and subsequently washed with distilled water. A single seed was placed in each pot and then covered with vermiculite. The pots were kept on trays and watered with autoclaved Hoagland solution (Hoagland and Arnon, 1938). Plants were grown before inoculation for 7days (wheat) or 14days (rice) at 25°C, 50% humidity, and a 16L:8D photoperiod (Supplementary Figures 1B–C).

### Diagnostic PCR and Real Time Quantitative PCR

Diagnostic PCRs were performed in a T-Professional-Gradient Thermocycler (Biometra). The reaction volume was 12.5μl containing 6.9μl ultrapure H_2_O, 1.25μl of 10x reaction buffer, 0.25μl 25mM MgCl_2_, 1.5μl 2mM dNTPs, and 1μl 10pmol/μl of each forward and reverse primer, 0.1μl of 5U/μl Taq DNA polymerase, and 1μl template. Ultrapure H_2_O was used as a negative control. For insect DNA samples acquired in section “Acquisition of the culturable symbiont from leaf litter”, the reaction volume was 12.5μl containing 5.36μl of Millipore H2O, 1.2μl 1x reaction buffer, 0.5μl 2.5mM MgCl_2_, 1.44μl 2mM dNTPs, and 1μl 10pmol/μl of each forward and reverse primer, 1μl 0.5U/μl Taq DNA polymerase, and 1μl template.

Quantitative PCRs (qPCRs) were conducted in a RotorGene®-Q cycler (Qiagen). The reaction volume was 10μl, consisting of 6μl ultrapure H_2_O, 2μl EvaGreen (Solis BioDyne), 0.5μl of each forward and reverse primer, and 1μl template. Standard curves were generated for absolute quantification of the templates. As a standard, we used a tenfold dilution series of the corresponding purified PCR product, including concentrations ranging from 1 to 10^−7^ng/μl according to Qubit® fluorometric measurements. For insect complimentary DNA (cDNA) samples acquired in section “Acquisition of the culturable symbiont from leaf litter”, the reaction volume was 25μl containing 6.5μl ultrapure H_2_O, 12.5μl SYBR Green Mix (Qiagen), 2.5μl of each forward and reverse primer, and 1μl cDNA template. Diagnostic PCR and qPCR cycle conditions can be found in Supplementary Table 1.

### Sanger Sequencing, Sequence Analysis, and Amplicon Cloning in *Escherichia coli*

PCR products were purified using the innuPREP PCRpure Kit (Analytik Jena-Biometra) following the manufacturer’s instructions, and the sephadex purification was carried out before Sanger sequencing. Samples were either sequenced mono-directionally using a capillary sequencer (ABI PRISM® 3130/ABI 3130xl Genetic Analyzer; Sanger et al., 1977) or by a commercial service (StarSeq, Mainz, Germany). The sequences were compared to corresponding references from Lv-StB and other symbiotic *B. gladioli* strains of *L. villosa* (Flórez et al., 2017; Waterworth et al., 2020). Low quality positions were manually curated and replaced with ambiguities.

Before Sanger sequencing of 16S rRNA fragments amplified with unspecific primers (Section: Bacterial Transmission From the Insect to Plants), these were cloned into *Escherichia coli* using the StrataClone PCR cloning Kit (Agilent) following the manufacturer’s instructions. These corresponded to samples from five wheat plants, three rice plants, and three soybean plants. Briefly, 100μl of the resulting transformation mixture was plated on LB–agar plates containing ampicillin (100mg/l), which had been treated with 40μl of 2% X-gal beforehand, and the plates were incubated at 37°C overnight. As a final step, white colonies were picked and amplified with the M13 forward and reverse primers (Supplementary Table 2). Amplicons of correct size were sent for Sanger sequencing. After filtering out samples with low base call quality, we obtained 41 sequences from the wheat treatment, 46 from rice, and 83 from soybean that were compared to known *B. gladioli* symbiont strain sequences.

### Bacterial Transmission From the Insect to Plants

To evaluate whether the symbionts can be transferred from the beetle to wheat (*Triticum aestivum*) and rice (O*ryza sativa*), plants were sowed in standard conditions as described in “Standard Sowing for Semi Sterile Plant Rearing.” Two groups per host plant (treatment and control) of 18 plants each were placed alternating a control and a treated individual, keeping conditions at 25°C, 50% humidity, and a 16L:8D photoperiod. A clip cage, first cleaned by rinsing with 70% ethanol and exposure to UV light for 30min, was attached to the oldest leaf of each plant and the cage was supported so that the leaf was not overloaded by its weight (Supplementary Figure 1D). An adult field-collected female beetle and a small water-drenched cotton ball were added to each cage in the treatment group. The cages in the control group contained no beetle but were otherwise treated identically. After 3days, the cages were removed, and plants were grown for 11 additional days. Individual beetles were stored in 70% EtOH at −20°C until the symbiont-containing glands were dissected. DNA was extracted individually along with five no template extraction controls using the Epicentre MasterPure™ kit and following the manufacturer’s instructions. Leaf tissue was collected from each plant in an area that was within the corresponding clip cage. RNA was extracted utilizing the Epicentre MasterPure™ kit following the manufacturer’s instructions and reverse transcribed using a QuantiTect® Reverse Transcription kit as indicated by the manufacturer. In addition, we analyzed cDNA from soybean (*Glycine max*) plants exposed to beetles as described previously (Flórez et al., 2017), but refining the analysis to the strain level. To assess the presence of *Burkholderia*, we targeted the 16S rRNA, gyrase subunit B and the trans-AT PKS lgaG genes (Supplementary Table 2). Furthermore, high-throughput amplicon sequencing of the bacterial 16S rRNA gene was carried out on the dissected symbiotic glands of each beetle and leaf tissue from the corresponding plant, as described in “High-Throughput Amplicon Sequencing on Bacterial 16S.”

### Bacterial Transmission From the Insect to Leaf Litter and Soil

To assess whether the *Burkholderia* bacteria from *L. villosa* can be transmitted to and survive on leaf litter, 12 groups of 5–6 field collected adult beetles were kept in rearing boxes. In addition, 10 boxes without beetles were treated identically and served as a control. Every second day, a piece of fresh and dried leaf of soybean (*G. max*), pea (*Pisum sativum*), and rape (*Brassica napus*) were placed in the boxes. After 30days of exposure, all leaves were collected from each box. Following homogenization, RNA was extracted from the leaf tissue. The obtained cDNA was analyzed *via* qPCR using the primers Burk16S_1_F and Burk16S_1_R (Supplementary Table 2).

To evaluate transmission to the environment in semi-natural conditions, the presence of *Burkholderia* bacteria was monitored in terrariums containing soil, live soybean plants, and leaf litter and set up as described in “Influence of the Environment in Symbiont Maintenance.” In a first assay, fresh leaves, withered leaves, and soil samples (six of each) were collected directly before placing 31 *L. villosa* adults, pupae, and larvae. Six weeks after introducing the insects, six fresh leaves, six withered leaves, and nine soil samples were collected and stored dry at −80°C. DNA was extracted using the DNeasy PowerSoil Kit (Qiagen) as indicated by the manufacturer. In a second terrarium assay aiming to evaluate the presence of live bacteria, six replicates of each sample type (withered leaves, fresh leaves, and soil) were collected before introducing 28 *L. villosa* of different life stages. After six weeks, six replicates of each sample type were collected. RNA was extracted using the RNeasy PowerSoil Total RNA Kit (Qiagen) following the manufacturer’s instructions and reverse transcribed into cDNA using a FIREScript RT cDNA Synthesis KIT (SolisBioDyne) as indicated by the manufacturer. All samples were analyzed for the presence of *B. gladioli* and specifically *Burkholderia* Lv-StB *via* diagnostic PCRs targeting the gyrB and lgaG genes using primer pairs gla-Fw/gyrB-Burk_R and LgaG_3_fwd/LgaG_4_rev (Supplementary Table 2) and subsequent Sanger Sequencing. Additionally, 10 leaf litter samples of soybean (*G. max*) and radish (*Raphanus* sp.) plants from a beetle collection site were analyzed. DNA was extracted with the Epicentre MasterPure™ (Epicentre Technologies) kit following the manufacturer’s instructions and screened *via* PCR targeting the 16S rRNA, gyrB, and lgaG genes (Supplementary Table 2) for the presence of the symbiotic bacteria. To verify the species of collected field plants, we used primers targeting the maturase K (matK) plastid gene and the ribulose-1,5-bisphosphate carboxylase/oxygenase large subunit (rbcL) plastid gene (Supplementary Table 2).

### Plant Infection by Culturable Symbionts

To investigate the interaction of culturable symbiotic *B. gladioli* bacteria with wheat and rice, plants of each species were inoculated with Lv-StA. After standard sowing as described in “Standard Sowing for Semi Sterile Plant Rearing,” 40 plants per species of similar height were selected and a leaf was wounded in the central area using a razor blade. Half of the plants per species were inoculated on the wound with 5μl of a 10^6^ cell/μl suspension of Lv-StA, and water was applied to the second half. After 21days (wheat) or 28days (rice), three tissue samples were taken from six plants of each group; the first from the original infection location (ori), the second from the next leaf developed after infection (new), and the third one from the stem between those two leaves (stem; Supplementary Figure 1E). The tissue samples were weighed, and RNA was extracted, reverse transcribed, and the 16S rRNA gene copy numbers were identified *via* qPCR, using the primers “Burk16S_StA-G_F” and “Burk3.1Rev.” (Supplementary Table 3). In addition, plant height was measured on the day of infection and every 7days over the course of the experiment, and leaf necrosis was monitored qualitatively. All plants were subsequently dried for 24h at 70°C, either completely or without the previously collected tissue samples, and their dry weight was measured using a Denver Instrument SI-64 balance to a precision of 0.1mg.

### Acquisition of the Culturable Symbiont From Leaf Litter

To obtain symbiont-free (aposymbiotic) individuals, *L. villosa* eggs were submerged in 95% ethanol for 5min and rinsed thoroughly with autoclaved water. Subsequently, they were sterilized in 12% NaClO for 30s and rinsed again with autoclaved water. Sterile PBS was then added to the eggs at a volume corresponding to 2.5μl per egg. To evaluate whether larvae that hatched from treated eggs were symbiont-free, a diagnostic PCR with *Burkholderia*-specific primers (Burk3.1fwd and BKH1434Rw, Supplementary Table 2) was performed on six individuals, confirming the absence of *Burkholderia*. Fresh and dried leaves of soybean (*G. max*), pea (*P. sativum*), and rape (*B. napus*) were inoculated with 20μl of a suspension containing 10^6^ CFUs of symbiotic *B. gladioli* Lv-StA in sterile tap water, by distributing the suspension on the leaves with a sterile paintbrush. As a control, leaves were smeared with sterilized tap water. Twenty (treatment) and 12 (control) symbiont-free *L. villosa* larvae at second or third instar were exposed to the leaves until completion of development (41±7days) under 14L:10D conditions. Temperature and humidity were monitored during the experiment. Inoculated leaves were placed inside larvae boxes every second day and sterile drinking water was replaced once a week. *L. villosa* individuals were collected shortly after reaching adulthood, surface sterilized in 70% ethanol and their sex was determined by dissecting each specimen. The paired accessory glands from females were separated carefully from the gut and the rest of the body. One of the two structures was kept at −80°C for testing the presence of bacterial symbionts by extracting DNA and RNA, with diagnostic and quantitative PCRs, respectively, targeting the 16S rRNA gene. The other half was kept in 70% ethanol for visualization of *Burkholderia* using FISH utilizing the probes Burk_16S-Cy3 and EUB338-Cy5 (Supplementary Table 3).

DNA and RNA were extracted using the MasterPure™ Complete DNA and RNA Purification Kit according to the manufacturer’s instructions. All solutions used for RNA extraction were prepared in Diethylpyrocarbonate (DEPC) treated H_2_O, and RNaseZap Decontamination Solution (Thermo Fisher Scientific, Waltham, United States) was used in order to avoid RNase contamination. The diagnostic PCRs were performed with the Lv-StA specific primer pair Burk16S_StA-G_F and Burk3.1Rev to evaluate horizontal acquisition of the provided strain, as well as with primers specific for Lv-StB (Burk16S_StB_F and Burk3.1Rev) to rule out background presence of the naturally dominant strain in the treated larvae (Supplementary Table 2). PCR amplifications were performed as described in “Diagnostic PCR and Real Time Quantitative PCR.” Reverse transcription of RNA was carried out with the QuantiTect® Reverse Transcription kit following the manufacturer’s protocol, and qPCR was conducted as described in “Diagnostic PCR and Real Time Quantitative PCR.” *Escherichia coli* K-12 and ultrapure H_2_O were used as controls.

### Influence of the Environment in Symbiont Maintenance

To assess whether the environment has an influence on the stability of the symbiosis, we set up two terrariums to simulate semi-natural conditions. Each container (80cm×120cm×35cm) was filled with a 2cm bottom layer of autoclaved vermiculite, followed by an 11cm layer of autoclaved soil, and then watered with 8L of tap water. Eleven soybean plants were planted in each. The containers were each covered with a cage (80cm×120cm×100cm) that allowed for complete illumination and ventilation. Temperature, humidity, and day-night cycle were kept equivalent to standard rearing.

Thirty-one field-collected *L. villosa* from different life stages (larvae, pupae, and adults) were initially introduced. After six weeks, six larvae were collected from different areas in the habitat, as well as three larvae from standard rearing containers (see Beetle collection and standard rearing). On week 9, seven additional larvae from the terrarium and 10 larvae from the standard containers were sampled. Samples were stored dry at −80°C until DNA of all samples was extracted using the DNeasy PowerSoil Kit (Qiagen). The second terrarium initially housed 27 field-collected individuals. In this case, 24 larvae were collected from the habitat six weeks after introducing the insects, alongside 19 larvae from standard containers, and stored identically. Larval DNA was extracted with the Epicentre MasterPure™ kit (Epicentre Technologies). All procedures were conducted following the manufacturer’s instructions. Diagnostic PCRs were carried out on the lgaG gene with subsequent Sanger sequencing as described above (Sanger sequencing, sequence analysis, and amplicon cloning in *E. coli*).

### High-Throughput Amplicon Sequencing on Bacterial 16S

For plant samples, cDNA generated as described in “Bacterial transmission from the insect to plants” was used. For female beetle glands, genomic DNA was obtained as also detailed in “Bacterial transmission from the insect to plants”, including five negative controls for DNA extraction. The V4 region of the 16S rRNA gene was sequenced by a commercial provider (StarSeq, Mainz, Germany) on an Illumina MiSeq platform with V3 chemistry, using primers 515F (Parada et al., 2016) and 806bR (Caporaso et al., 2011, 2012), double indexing, and a paired end approach with read length of 300nt, as well as 25% PhiX to balance the composition of bases. As sequencing controls, two no template samples and one DNA standard sample (ZymoBIOMICS™ Microbial Community DNA Standard) were included. Demultiplexing was carried out using bcl2fastq2 including the generation of fastq files for indexes and allowing for zero mismatches in the barcodes. Amplicon sequence variants (ASVs) were inferred using the R package DADA2 (Callahan et al., 2016) with default parameters including dereplication and chimera removal. Reads were trimmed to lengths of 250 and 140nt for forward and reverse reads, respectively. Taxonomy was assigned using the pre-trained classifier Silva 132 (Quast et al., 2013; Yilmaz et al., 2014) with subsequent removal of reads classified as chloroplast or mitochondria. To elucidate strain-level diversity, all 52 bins assigned to the family Burkholderiaceae were further analyzed. The representative sequence of each bin was blasted against a local database containing Lagriinae-associated *Burkholderia* strain sequences and every representative sequence that had a pairwise identity of over 99% to a sequence in the database was assigned accordingly.

During data analysis, we detected presumable misassignments in sequencing indexes. To mitigate false positives, we used the python script unspread.py (Larsson et al., 2018). The spreading rate (0.0002) was calculated as the number of Lv-StB reads in experimental control samples divided by the median of Lv-StB reads in samples with high read numbers (beetle glands) in each corresponding column and row on the sequencing plate.

### Fluorescence *in situ* Hybridization

Fluorescence *in situ* hybridization was performed to localize *Burkholderia* in plant tissues as well as in dissected beetle glands. The samples were individually fixed in Carnoy’s solution (67% ethanol, 25% chloroform, and 8% glacial acetic acid) and dehydrated in an n-Butanol series of ascending concentration (30, 50, 70, 80, 90, and 96%). The samples were then embedded in Technovit 8100 (Heraeus Kulzer) following the manufacturer’s instructions. Histological sections of 8μm thickness were cut with a glass knife on a Leica RM 2245 microtome and transferred to glass slides. For the FISH hybridization mix, fluorescently labelled oligonucleotide probes (Supplementary Table 3) were diluted 1:20 in hybridization buffer, and 5μg/ml DAPI was added for host cell counterstaining. The hybridization buffer consisted of 80% dH_2_O, 18% 5M NaCl, and 2% 1M Tris/HCl (pH=8) with an additional 1μl 10% SDS per 1ml. Each slide was treated with 100–150μl hybridization mix and covered with parafilm. The slides were placed on a moist paper towel in a closed box and hybridized for 90min at 50°C. Subsequently, slides were transferred into wash buffer and incubated at 50°C for 20min. The wash buffer consisted of 95% dH_2_O, 2% 5M NaCl, 2% 1M Tris/HCl (pH=8), and 1% 0.5M EDTA with an additional 1μl 10% SDS per 1ml. Following another washing step in distilled water at 50°C for 20min, slides were briefly air dried in the dark, a small drop of VectaShield® was applied to each sample and a cover slip was added. Samples were observed under an Axio Imager.Z2 epifluorescence microscope (Zeiss) equipped with an ApoTome.2. Pictures were taken with an Axiocam 506 monochromatic camera (Zeiss) and processed with AxioVision software (Lite Edition 4.8.1, Zeiss).

## Results

To better understand the role of the plant environment in the *L. villosa – Burkholderia* symbiosis, we first tested the potential for the symbiotic bacteria to be transmitted to and survive in live plants or leaf litter (sections “Transmission of the beetle symbionts to plants,” “Culturable *B. gladioli* Lv-StA survive and spread in plants after inoculation,” and “Transmission of beetle symbionts to soil and leaf litter”) and then assessed their potential for being acquired by the beetle from the plant environment (“Acquisition of a culturable *Burkholderia* symbiont from the environment”). Finally, given our previous observations on symbiont loss in controlled laboratory conditions, we evaluated whether a semi-natural environment containing live plants, soil, and leaf litter contributes to the overall stability of the symbiosis (“The plant environment sustains the symbiosis with *Burkholderia*”).

### Transmission of the Beetle Symbionts to Plants

To evaluate whether *L. villosa* beetles can transmit their bacterial symbionts to plant hosts, we screened for the presence of *Burkholderia* in leaves from rice and wheat plants that were in contact with the beetles. As detailed below, we used cDNA-based diagnostic PCRs and Sanger sequencing, as well as 16S rRNA amplicon sequencing of both the leaves and the accessory glands dissected from the individual adult females that were exposed to each plant.

#### *Burkholderia* Screening Based on Multiple Gene Markers

**Figure 1:**
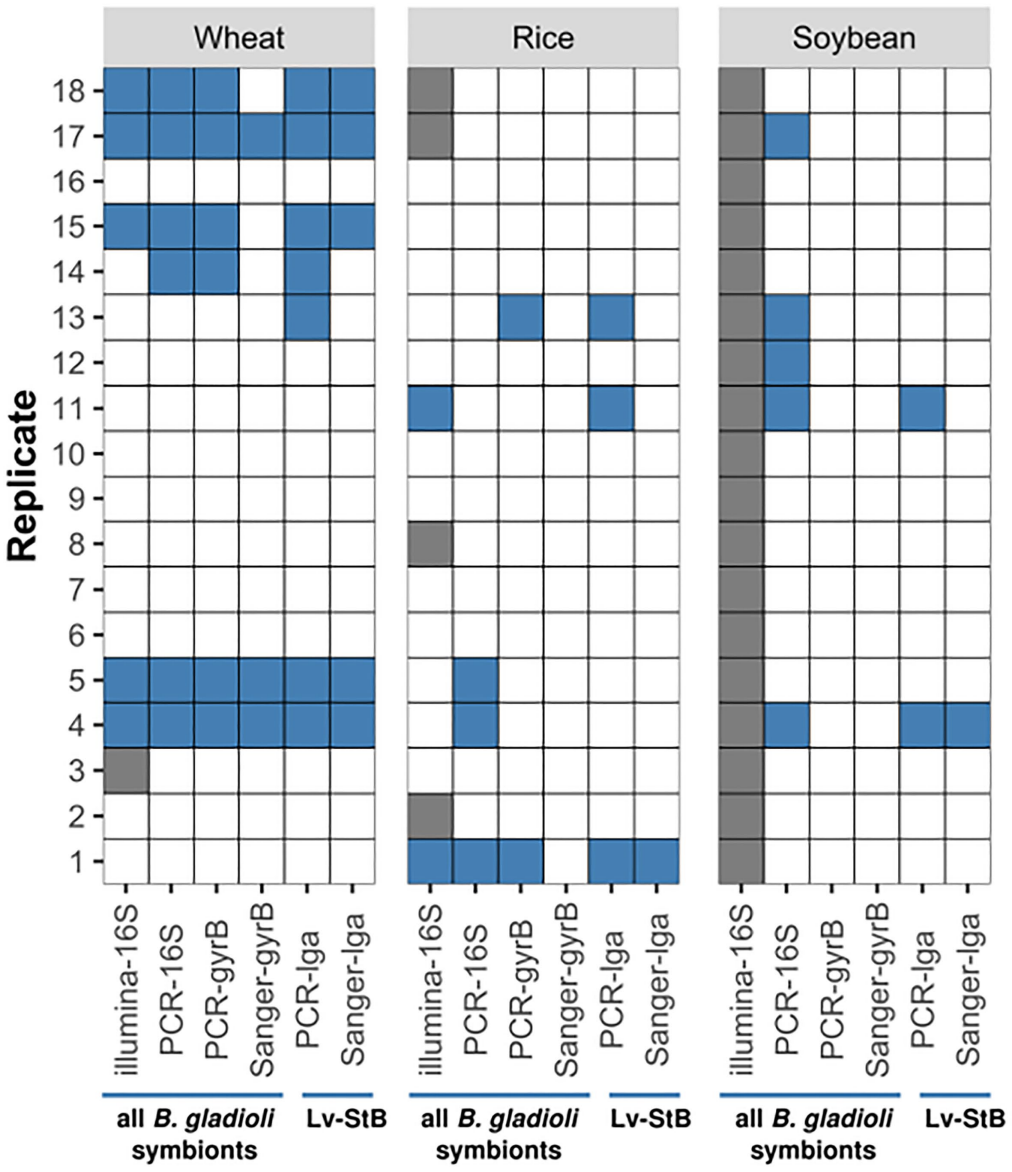
Transmission of *Burkholderia* strains from *Lagria villosa* adults to wheat, rice, and soybean plants. Results were obtained with diagnostic PCRs using *Burkholderia*-specific primers targeting the 16S rRNA and gyrB genes, or *Burkholderia* Lv-StB – specific primers targeting the lgaG gene. Sanger or Illumina sequencing was carried out for the obtained amplicons. The *B. gladioli* strains targeted by each method are indicated below the blue horizontal lines. Blue filled tiles represent a positive indication of *Burkholderia* presence, empty tiles indicate no sign of *Burkholderia* presence, and grey tiles indicate the method was not applied on that sample. Occasionally, a signal was found *via* PCR but sequencing was unsuccessful. All controls (*N*=18 for wheat and rice, respectively, *N*=9 for soybean) were negative across all methods.

To determine whether and which *Burkholdera* strains were transmitted to rice and wheat plants, as well as soybean plants, we carried out PCRs on cDNA targeting the genes encoding for the 16S rRNA, the DNA gyrase subunit B, and a trans-AT polyketide-synthase specific for the production of lagriamide (lgaG). Lagrimide is exclusively synthesized by the dominant symbiont strain Lv-StB (Flórez et al., 2018; Waterworth et al., 2020). Additionally, we carried out Sanger sequencing of the gyrB and lgaG amplicons to confirm that the sequences indeed originated from Lv-StB. We used reverse-transcribed RNA of plant tissues collected 11 or 12days after exposure to the beetles, aiming to assess the presence of live cells. Across all three different plant species, we found evidence for symbiont presence in individual plants exposed to *L. villosa* beetles. Given that amplification was not fully consistent across different genes for a given sample, we considered a positive result in two or more genes, including sequencing confirmation, to be plants that were very likely infected. Based on this assessment, Lv-StB was detected with high certainty in one rice and one soybean individuals out of 18 plants, respectively, while in wheat, the infection rate was of five out of 18 individuals (Figure 1). However, a less conservative estimation suggests that between five and seven individuals per species, i.e., close to one third of the plants, were infected. No Lv-StB bacteria were detected in any of the 45 unexposed control plants (*N*=18 for wheat and rice, *N*=9 for soybean).

#### *Burkholderia* Screening Based on Illumina 16S rRNA Amplicon Sequencing

We carried out 16S amplicon sequencing to complement the previous approach in assessing *Burkholderia* infection in the exposed leaves, and to evaluate the presence of Lv-StB and other associated bacteria in the glands of each corresponding beetle. After quality trimming and filtering, merging of pairs and chimera removal, we obtained between 29,259 and 153,209 reads per sample, with an average of 67,309 reads. A low number of Lv-StB reads was present across a majority of the library presumably due to crosstalk associated to index misassignments, which can occur in multiplexed sequencing libraries (Wright and Vetsigian 2016; Larsson et al., 2018). To overcome this issue, a correction was applied following the previously described “unspread” method (Larsson et al., 2018). After this adjustment, the samples contained between 29,087 and 153,155 reads, and 67,198 on average. Particularly in the plant samples, a considerable fraction of these reads corresponded to chloroplasts, which were filtered out from the data set along with mitochondria, obtaining minimum 71 and maximum 152,954 reads per sample, 37,080 on average, and a total of 1,028 ASVs (Supplementary Table 4). Samples that retained less than 50 reads were not considered further. Since the aim of the study was not to provide profiles of the plant microbial community, but rather to confirm the presence of our target strain Lv-StB, we went further with this amount of reads in plant samples despite the considerably low threshold. For the same reason, the data from rice and wheat should not be considered representative regarding the overall community composition and diversity.

**Figure 2:**
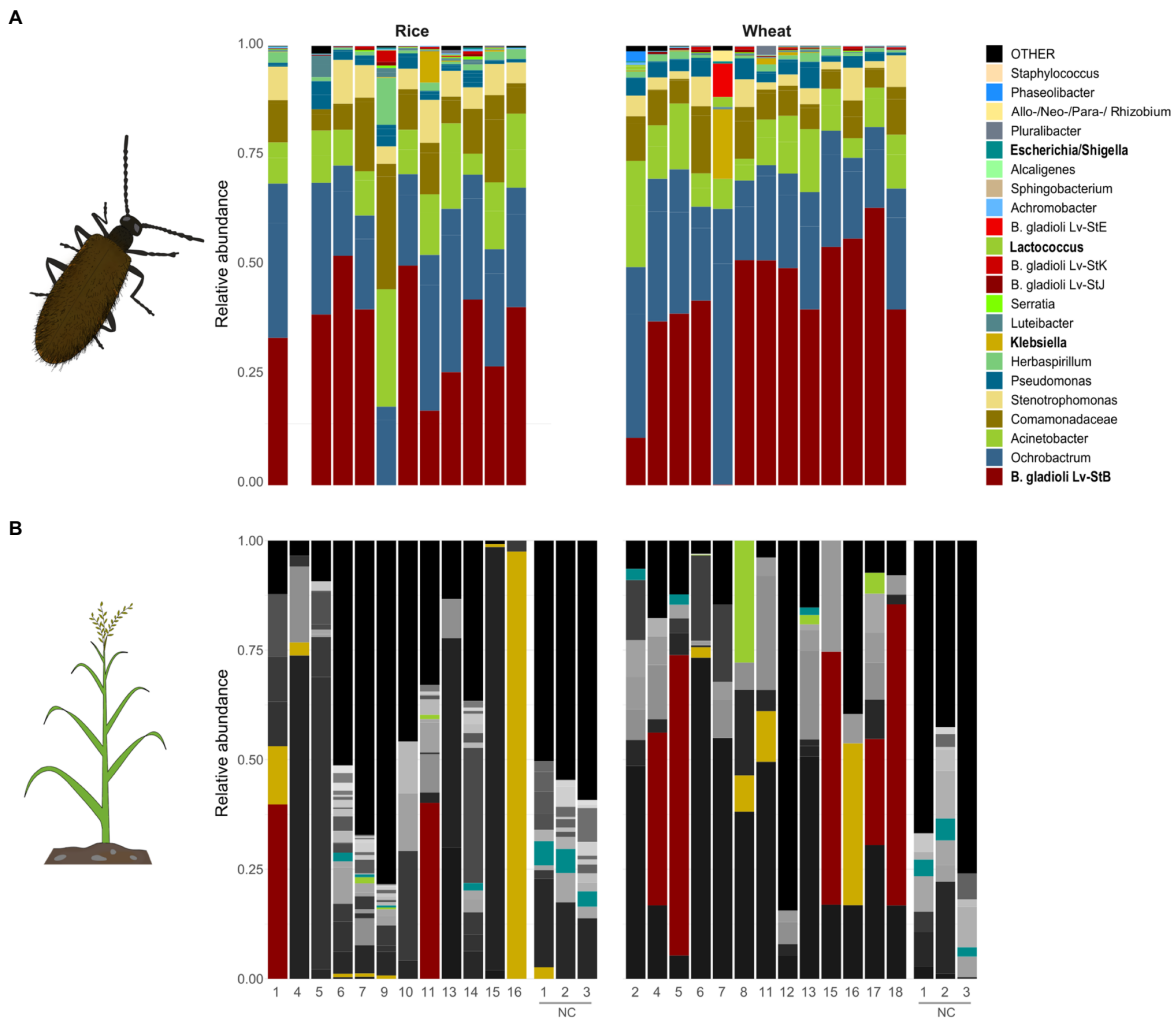
Bacterial community composition in **(A)** glands of *L. villosa* beetles and **(B)** plant tissue that was exposed to the corresponding beetles, excluding samples that did not meet the threshold after chloroplast sequence removal. The relative abundance of bacterial amplicon sequence variants (ASVs) assessed at genus level by DADA2 analysis on the V4 region of the 16S rRNA gene is represented. Only the 30 most abundant bacterial ASVs for each corresponding sample type (glands or plants) are shown, and the remaining ASVs are grouped as “OTHER.” Beetle-associated ASVs are highlighted in color, and ASVs only associated to plants are depicted in grey. Genera names highlighted in bold correspond to identical ASVs in both gland and plant samples. NC = Negative control pools from plants that were not exposed to beetles.

Most of the analyzed beetle glands harbored *B. gladioli* bacteria, that is 92.3% (12/13) of the insects placed on rice plants and 94.1% (16/17) of those on wheat plants. As expected, Lv-StB was dominant among *B. gladioli* strains, and only minor abundances of three other strains (Lv-StE, Lv-StK, and Lv-StJ) were occasionally present in the beetles (Figure 2A). The bacterial screening in the plants confirmed that *Burkholderia* is occasionally transmitted to rice and wheat plants (Figure 2B). In the unspread corrected data, Lv-StB was not part of the community in unexposed control plants that were sequenced as pools (Supplementary Figures 2, 3). By contrast, the symbiotic Lv-StB was found in 14.3% (2/14) of the rice plants and in 29.4% (5/17) of the wheat plants that had been in contact with beetles (Figure 1). Even when evaluating the uncorrected sequence data, which presumably still includes reads assigned to false samples due to cross-talk, results confirm that Lv-StB read counts were significantly higher (Mann-Whitney U test, *n*=7, *n*=35, *p*<0.001) for samples where the strain is present according to diagnostic PCR and Sanger sequencing tests (median read number=119) than for samples where the bacterium was not expected (median read number=0). This indicates that the overall conclusion of transmission potential is supported by the data independent of the quantitative correction.

According to our 16S rRNA amplicon sequencing data, no other bacteria besides *Burkholderia* were frequently transmitted to the plants when analyzing the 30 most abundant ASVs in the beetle glands (Supplementary Data 1). However, there may be occasional exchange of non-*Burkholderia* bacteria in either direction, or an indirect impact of beetle exposure on the local bacterial community in the leaf. Among the taxa that are predominant in both beetles and plants, only ASVs identified as *Klebsiella*, *Lactococcus*, and *Escherichia/Shigella* were sporadically shared between the beetle and the corresponding plant individual, in addition to Lv-StB (Figure 2; Supplementary Figure 4).

### Culturable *B. gladioli* Lv-StA Survive and Spread in Plants After Inoculation

About 21days after manual infection of wheat plants with the culturable symbiont Lv-StA, we found significantly higher cDNA copy numbers of the 16S rRNA gene in the originally inoculated leaf in treated plants than in uninfected control individuals. There was some background amplification in a few of the uninfected controls, but the differences with the infected treatment were highly siginficant (Kruskal Wallis: *χ*^2^=11.39, *N*=6, df=2, and *p*<0.005; *Post hoc* Dunn’s – Test: *z*=2.87; *p*<0.005) (Supplementary Figure 5). An analogous result was found for rice plants 28days after the infection. Again, we found higher titers within the originally inoculated leaf in treated plants than in control plants (Kruskal Wallis: *χ*^2^=11.39, *N*=6, df=2, and *p*<0.01; *Post hoc* Dunn’s – Test: *z*=3.06; *p*<0.005) (Supplementary Figure 6) We have included all Ct values and calculated copy numbers in Supplementary Data 2.

Lv-StA can also translocate to younger leaves. This is indicated by the higher cDNA copy numbers of the symbionts compared to unexposed controls in wheat plants (Kruskal Wallis: *χ*^2^=7.14, *N*=6, df=2, and *p*<0.05; *Post hoc* Dunn’s – Test: *z*=2.34; *p*<0.05) as well as in rice plants (Kruskal Wallis: χ^2^=7.14, *N*=6, df=2, and *p*<0.05; *Post hoc* Dunn’s – Test: *z*=2.83; *p*<0.005) (Supplementary Figures 5, 6).

### Transmission of Beetle Symbionts to Soil and Leaf Litter

**Figure 3:**
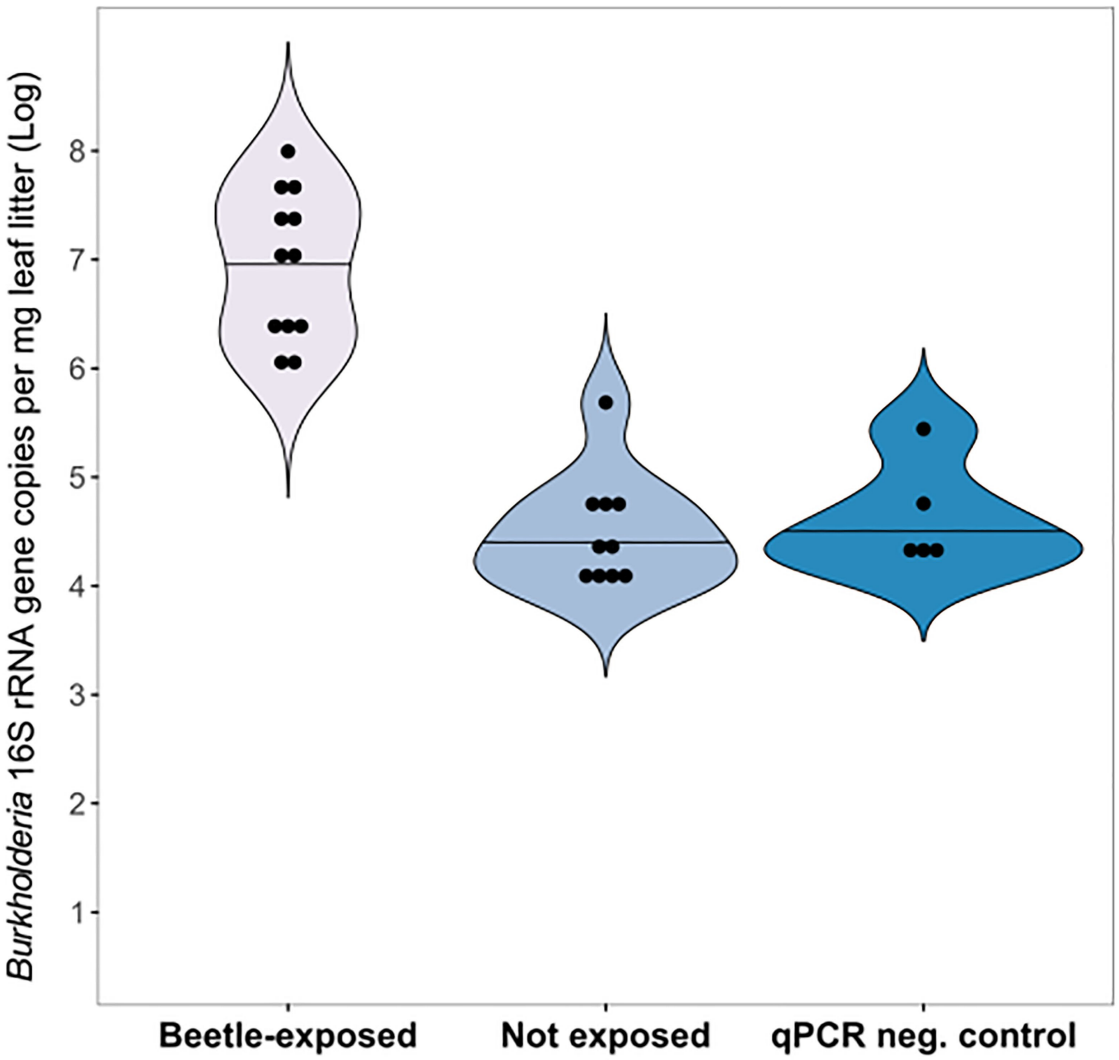
Symbiont transmission from beetles to leaf litter. *Burkholderia* 16S rRNA was detected in leaf litter samples exposed to adult *L. villosa* beetles (*N*=12) in significantly higher copy numbers than in unexposed leaves serving as a control (*N*=10; Wilcoxon Rank Sum test, *p*<0.001), which resembled quantitative PCR (qPCR) negative controls (NC). Dots within the violin plot represent individual samples and horizontal lines indicate the median. NC includes sterile water (3x) and *Escherichia coli* genomic DNA (2x).

In addition to infections on live host plants, we examined whether the symbionts could be exchanged with leaf litter and soil in the environment. Dry leaves that had been deliberately exposed to field-collected *L. villosa* female beetles carried on average 3×10^7^ *Burkholderia* 16S rRNA gene copies based on the quantification of cDNA per mg leaf litter (Supplementary Data 2). These titers were significantly higher than those detected in unexposed leaves, which fell in the range of no template controls in the qPCR (Wilcoxon Rank Sum test, *p*<0.001, Figure 3). However, the specific identity of the *Burkholderia* strain(s) was not established in these samples. In a semi-natural environment including live plants and soil that were exposed to *L. villosa* beetles, DNA of Lv-StB was detected in fresh leaves (33.3%; 2/6), leaf litter (16.7%; 1/6), and soil (22.2%; 2/9) *via* lgaG-specific diagnostic PCR and subsequent Sanger sequencing. However, an RNA-based assessment from a subsequent experiment did not confirm the long-term survival of Lv-StB symbionts in leaves and soil within the terrarium. When evaluating environmental samples taken from beetle collection sites, Lv-StB DNA was found in leaf litter in three of 10 soybean and radish samples. However, whether this corresponds to live or dead cells is unknown.

### Acquisition of a Culturable *Burkholderia* Symbiont From the Environment

**Figure 4:**
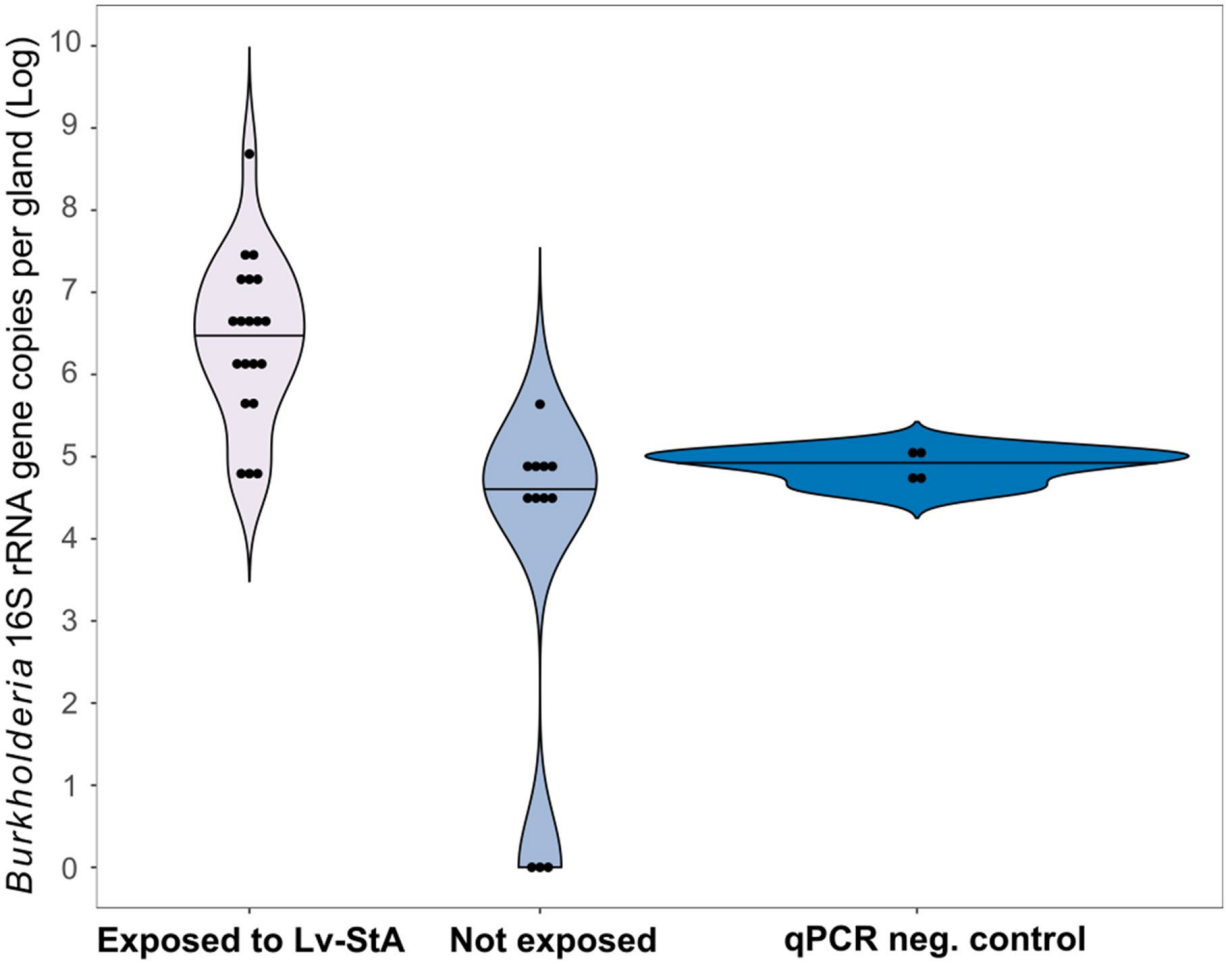
Environmental acquisition of *B. gladioli* Lv-StA by *L. villosa* evaluated *via* qPCR. Abundances of Lv-StA in single glands of adult *L. villosa* females exposed as aposymbiotic larvae to the bacteria are shown. Detected gene copy numbers of Lv-StA 16S rRNA are significantly higher in individuals that were exposed to infected leaves (*N*=20) than in non-exposed control insects (*N*=12; Wilcoxon Rank Sum test, *p*<0.001). Titers in non-exposed beetles were on par with or below qPCR NCs, which include no-template samples (3x) and *E. coli* genomic DNA (1x). Dots within the violin plot represent individual samples and lines indicate the median.

**Figure 5:**
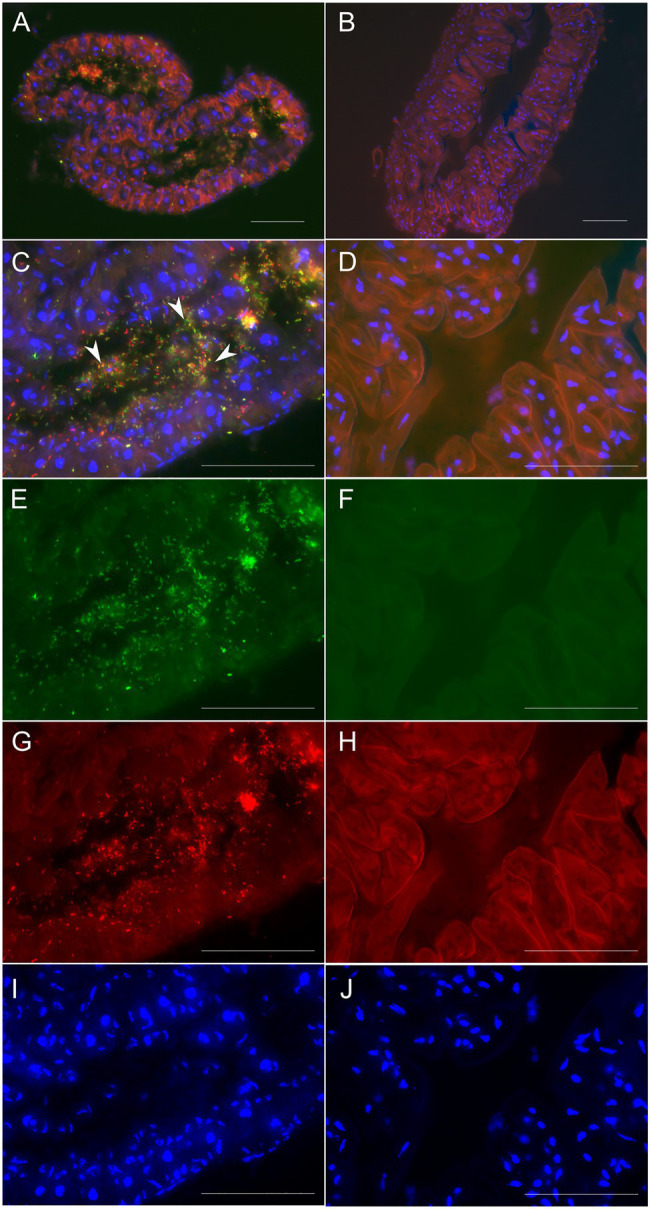
Micrographs showing environmental acquisition of *B. gladioli* Lv-StA by *L. villosa* or absence in an unexposed control. **(A,C,E,G,I)** Lv-StA presence in cross-sections (8μm) of the accessory glands of an adult *L. villosa* that had been exposed to Lv-StA during the larval stage. **(B,D,F,H,J)** show an aposymbiotic control individual. In **(A)** and **(C)**, symbiont cells are represented in yellow and white arrowheads denote exemplary symbiont cells. Fluorescent signals in individual channels correspond to **(E,F)** *Burkholderia* specific labeling in green (Burk_16S-Cy5), **(G,H)** general eubacteria labelled in red (EUB338-Cy3), and **(A–D,I,J)** nucleic acids unspecifically labeled in blue (DAPI) showing host cell nuclei. The scale bars represent 50μm. For clarity, **H** shows autofluorescence from the host tissues in the Cy3 channel.

Aposymbiotic individuals in the larval stage that were exposed to leaf litter smeared with Lv-StA picked up the symbiont and accommodated the bacteria up to the adult stage in 70% (14/20) of the cases. We considered a sample as infected only if it had a positive result in the diagnostic PCR and was above 1×10^6^ *Burkholderia* 16S rRNA gene copies based on the cDNA-based qPCR (Supplementary Data 2). The unexposed control group showed weak signs of *Burkholderia* in three of the 12 samples, although all were below 1×10^6^ *Burkholderia* 16S rRNA gene copies. There were in general significantly higher titers of bacteria in symbiont-exposed individuals compared to unexposed counterparts as measured *via* qPCR on cDNA. Additionally, the melting curves suggest that some unspecific amplification occurred in the controls although we refrained from applying any corrections. Despite this conservative approach, the difference between treatments was highly significant (Wilcoxon Rank Sum test, *p*<0.001; Figure 4). FISH using *Burkholderia*-specific and general eubacterial probes on histological sections of adult females that had been exposed to Lv-StA as larvae revealed that the *Burkholderia* bacteria successfully colonized the accessory glands of the female reproductive system in two out of two analyzed individuals, whereas none of two inspected individuals of the control group showed presence of *Burkholderia* (Figure 5). Notably, if in natural conditions vertical transmission of Lv-StB and other bacteria in the maternal secretions is successful, the probability of incorporating horizontally acquired strains might be reduced. Therefore, we expect that the rate of horizontal transmission in the field is likely lower than what we observe here.

### The Plant Environment Sustains the Symbiosis With *Burkholderia*

*L. villosa* are more likely to keep their symbionts across generations in an environment that resembles natural living conditions with living plants and soil, than in plastic containers lacking substrate and with a controlled supply of harvested leaves and water (Terrarium 1: *χ*^2^=9.8462, df=1, *N*=26, and *p*<0.005; Terrarium 2: *χ*^2^=7.9922, df=1, *N*=43, and *p*<0.005). Considering all evaluated individuals, 86.5% of first lab generation larvae retained their symbionts in semi-natural terrarium conditions whereas only 31.3% of larvae from the same generation larvae kept in plastic containers maintained the Lv-StB symbiont as assessed by diagnostic PCR and Sanger Sequencing (Supplementary Figure 7).

## Discussion

**Figure 6:**
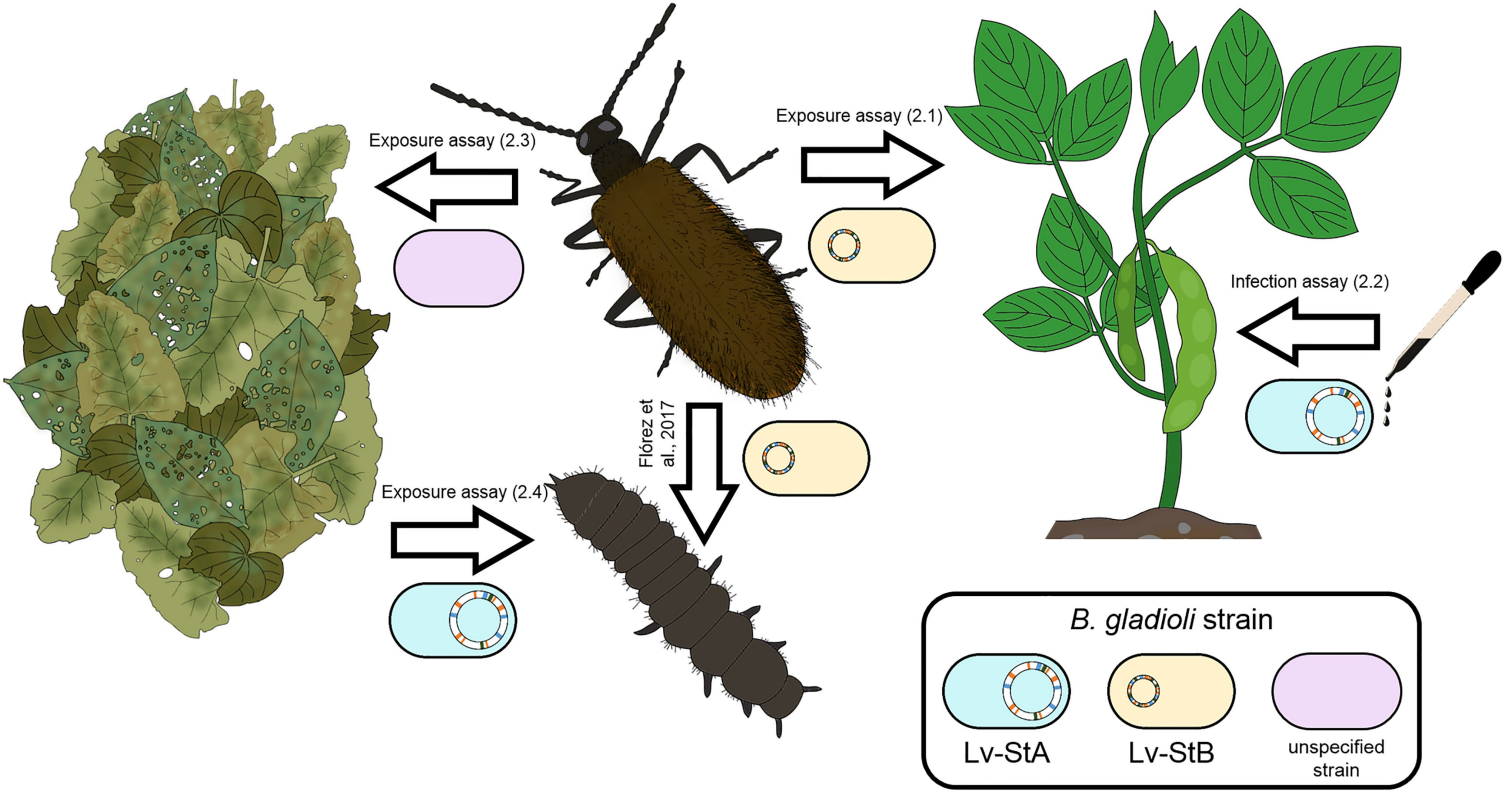
Overview of demonstrated transmission routes in the *L. villosa – Burkholderia* symbiosis. The beetles can transmit the reduced-genome symbiont Lv-StB vertically, as well as horizontally to plants. In addition, they can transmit unidentified *B. gladioli* strain(s) to leaf litter. Lv-StA, the culturable strain without signs of genome erosion, is capable of infecting and surviving in different plant hosts and can be acquired from the leaf litter by the insect. The numbers in parenthesis indicate the section of the manuscript, or the published reference, in which evidence for each transmission route is provided.

Here, we demonstrate that bacterial symbiont strains of *L. villosa* beetles with starkly different genome sizes and host-dependence can be transmitted to live plants. In addition to the established route for vertical transmission, the beetles can acquire *Burkholderia* symbionts from the environment and are more likely to retain the naturally dominant symbiont strain if continuously exposed to soil and plants (Figure 6). This indicates that the beetle-symbiont association is partially open to exchange with the environment and there is likely a mixed-mode transmission of at least two symbiont strains.

### Transmission Routes of *Burkholderia* Strains in *Lagria villosa*

A mixed-mode transmission confers potential benefits for the host and is in line with the notion that exclusive vertical or horizontal transmission are likely the exception rather than the rule (Ebert, 2013). While the colonization of offspring is guaranteed for each generation through highly reliable vertical transmission routes, additional horizontal acquisition can be an effective way to pick up symbionts early in development if the vertical transmission route is less secure. Also, occasional horizontal symbiont acquisition might be advantageous for the host to rapidly adapt to evolutionary challenges like unstable environments or fluctuating occurrence of natural enemies (da Costa and Poulsen, 2018). Similarly, a mixed transmission mode might facilitate the association with multiple symbiont strains with a broader metabolic spectrum. This is plausible in the *Lagria* – *Burkholderia* symbiosis, given that the antifungal compound produced by Lv-StB, lagriamide, is not produced by any of the related strains associated to *L. villosa*, including Lv-StA. In turn, Lv-StB does not synthesize any of the so far identified bioactive compounds produced by Lv-StA, some of which are also antibacterial (Flórez et al., 2017, 2018; Waterworth et al., 2020). Therefore, these distinct strains may be able to protect the offspring against a wider variety of pathogens in the environment. It is important to note, however, that control mechanisms regulating the composition of the community are likely important for the stability of the association in a system that is partially open to the environment. Such means for regulation might come from the host, the bacteria, or both, as has been described in other symbioses involving horizontal transmission (Ohbayashi et al., 2020).

Maintaining the ability to leave the insect host and switch between multiple hosts can also be beneficial for the bacterial partner. The food plant can be a provisional nutrient-rich environment, or might serve as a refuge when the insect host is scarce, even if not an optimal environment for insect-adapted strains (Frago et al., 2012; Tago et al., 2015). Alternatively, some strains might be more closely affiliated to the plant and use the insect as a vector. In general, the opportunity for multiple transmission routes is expected to be beneficial for the bacteria unless there are significant trade-offs. The tendency to engage more strongly in one mode or the other is known to be plastic in other symbionts, and might depend on both the environment and host-associated factors (Ebert, 2013). Importantly, the possibility for adjusting the transmission mode ratio provides the opportunity to more flexibly respond to selective pressures (Ebert, 2013). Additionally, horizontal transmission ameliorates some of the negative consequences of strict vertical transmission on the symbiont, i.e., lack of recombination, subsequent accumulation of deleterious mutations, and gene loss facilitated by a small effective population size, particularly during transmission (Moran, 1996; Fisher et al., 2017). Consequently, mixed transmission might offer advantages to both the *L. villosa* beetles and the associated *Burkholderia*, although the fitness benefits of horizontal transmission remain to be confirmed for the predominant symbiont Lv-StB. Based on our screening experiments, it is unlikely that Lv-StB is present in high titers or reproduces rapidly in live plants. However, the specific detection of LvStB-RNA in rice, wheat and soy plants more than a week after contact with the beetles suggests that this strain can at least survive for some time in a plant host. This is supported by the presence of Lv-StB 16S rRNA gene sequence reads in plants exposed to beetles, despite the low number of total reads in the cDNA-based amplicon profiles. Based on the infection rates in this assay, it is possible that the chances of transmission and/or survival of *Burkholderia* differ between plant species, although the mechanistic basis of this difference is not known. Taken together, these results indicate that Lv-StB has the potential for horizontal transmission *via* plants, but the contribution of this route to the long-term success of the symbionts remains to be elucidated in future studies.

Mixed-mode transmission might represent a long-term stable strategy or an intermediate stage in evolution from environmental acquisition to vertical transmission. In the oriental chinch bug, for example, *Burkholderia* symbionts housed in gut crypts are regularly acquired from the environment, as also occurs in multiple other bug species of this and related superfamilies (Kikuchi et al., 2010; Itoh et al., 2014). However, in this species, close to a third of the nymphs take up the symbionts from the eggs suggesting a nascent vertical transmission route (Itoh et al., 2014). In *L. villosa*, it is likely that Lv-StB is at a more advanced stage in this process and its ability to survive in the plant, at least temporarily, is a remnant of a former lifestyle. We base this assumption on the fact that, compared to the other symbiotic *B. gladioli* strains in this beetle (Waterworth et al., 2020), as well as other free-living and host-associated *Burkholderia* bacteria (Beukes et al., 2017; Kaltenpoth and Flórez, 2020), Lv-StB has a considerably reduced genome and shows signs of increased host dependence. The strain is lacking a large proportion of genes involved in primary metabolism, as well as homologous recombination and DNA repair (Waterworth et al., 2020), which is in line with the process of genome erosion observed in symbionts with predominant vertical transmission (Muller, 1964; Moran et al., 2008; Fisher et al., 2017). Lv-StB also lacks genes for motility that are present in its closest relatives (Waterworth et al., 2020), which might limit transitioning between hosts unless external mechanisms aid in dispersal and colonization. Even though Lv-StB has lost many genes involved in primary metabolism, it still encodes a number of transporter genes that could facilitate nutrient uptake from the host and/or other microorganisms (Waterworth et al., 2020). Presumably, a key moment in the evolution of this symbiosis was the acquisition *via* horizontal gene transfer of the hybrid *trans*-AT PKS/NRPS biosynthesis gene cluster for the production of lagriamide in Lv-StB, which likely provides a fitness advantage for the host due to its antifungal activity (Flórez et al., 2018). Thereby, a more consistent association to this strain was potentially reinforced, enhancing vertical transmission and the associated genome erosion (Waterworth et al., 2020).

Other symbiotic *B. gladioli* strains, however, are not highly dependent on the beetle host and can infect several plants reaching different plant tissues, albeit in low numbers. The culturable strain Lv-StA was able to sustain an infection for at least 38days in soybean plants, and at least 21days in wheat and rice plants after manual inoculation (Supplementary Figures 5, 6). These observations, along with the considerably larger genome size of this strain (8–8.5Mb) and the only occasional occurrence of Lv-StA in field collected *L. villosa* beetles (Flórez and Kaltenpoth, 2017), are in line with a predominantly free-living or plant-associated lifestyle.

The specific means of horizontal transfer remains unclear and might be a byproduct of host behavior. Since the symbiont-housing glands of adult *L. villosa* females have direct openings to the exterior adjacent to the ovipositor, it is possible that these come into contact with the environment during egg-laying or when the ovipositor is partially protracted, which happens regularly in these beetles. Horizontal spread of symbionts to a plant during oviposition has been reported for example in fruit flies, where gut symbionts are transmitted to the plant and subsequently support the larvae by promoting decay of the plant tissue (Behar et al., 2008). In *L. villosa* larvae, the symbionts are housed in three dorsal cuticular invaginations (Stammer, 1929; Flórez et al., 2017) that might allow for exchange of bacteria with the soil and/or leaf litter. Accompanying these behavioral and morphological host traits, it is likely that at least some of the symbiotic strains evolved mechanisms that facilitate horizontal infection of the beetle. Regarding non-Lv-StB strains and non-*Burkholderia* bacteria present in the beetle female glands, these are generally not detected in plants after exposure to the insects. This might be associated to the low abundance and prevalence in the beetle, although occasional transfer in either direction cannot be ruled out. Furthermore, it is possible that not only entering the host glands, but also outcompeting other strains during the process of transmission and establishment is relevant, as has recently been shown for gut crypt-colonizing *Burkholderia* symbionts of stinkbugs (Itoh et al., 2019).

### The Soil and Plant Environment Positively Influences Maintenance of the Symbionts

The reason underlying symbiont loss under lab conditions by *L. villosa* – as well as *Lagria hirta* (Flórez and Kaltenpoth, 2017) – is not yet fully understood. It is worth noting that the symbiosis with *Burkholderia* is facultative for the beetle host, and infection rates of 100% are not necessarily expected nor observed in the congeneric species *L. hirta* (Flórez and Kaltenpoth, 2017). However, our results show that the symbionts are more likely to be maintained if the insects are kept in an environment with soil, leaf litter, and live plants rather than in cages only containing leaves and water. The semi-natural environment possibly serves as a source of bacteria, which is in line with our findings on the transfer from and to plants, as well as the potential presence of symbiotic *Burkholderia* in the field. Therefore, the maintenance of the *Burkholderia* – *L. villosa* symbiosis might be at least partially dependent on horizontal symbiont acquisition. Also, abiotic conditions potentially affecting transmission, like humidity or a specific microclimate, could be more suitable for symbiont persistence in the semi-natural setup. The more natural environment might additionally offer improved nutritional provisions for the beetle that indirectly support symbiont maintenance in the host. While the presumably lower exposure to pathogens in the frequently cleaned rearing boxes could reduce the pressure on maintaining costly defensive symbionts (Vorburger and Gouskov, 2011; Oliver et al., 2014; Polin et al., 2014), the observed loss occurs within a single generation and low pathogen pressure is thus unlikely to be the main driving factor.

### Conclusion and Outlook

We show that the reduced genome symbiont *B. gladioli* Lv-StB in *L. villosa* beetles can be transferred to plant material in addition to being vertically transmitted. The long-term persistence and consequences for the symbiont are however unknown. Our findings also demonstrate that a closely related strain with low host dependence can be exchanged with the environment. Together, these results suggest that a mixed-mode transmission occurs in the *Lagria* – *Burkholderia* symbiosis, maintaining a dynamic system, with the plant environment likely playing a crucial role in the stability of the association.

In some animal hosts – including many insects – a symbiotic association with specific microbes progresses toward an increasingly dependent and often exclusive partnership. However, many others including *Lagria* beetles sustain interactions that retain moderate flexibility. This might be stabilized by the potential advantages of a dynamic interaction, or could represent a snapshot of an ongoing process of co-evolution. In particular, the *Lagria-Burkholderia* symbiosis is a useful system to better understand the ecological and evolutionary drivers of symbiosis between herbivorous insect hosts and plant-associated bacteria, as well as the maintenance of strain diversity in defensive symbiosis.

## Data Availability Statement

The bacterial 16S rRNA gene amplicon datasets generated for this study are publically available in the NCBI Sequence Read Archive (SRA) under the BioProject with accession number PRJNA739268.

## Author Contributions

JW, PG, MK, and LF conceived the study and designed the experiments. JW was responsible for plant infection and beetle to plant transmission experiments, as well as amplicon sequencing analysis. PG carried out the transmission experiments from and to leaf litter, as well as FISH on beetle glands. DK was in charge of terrarium maintenance and the corresponding bacterial screening. LF and MK provided supervision on the experimental design and data analysis. JW and LF drafted the manuscript and all authors participated in editing the final version. All authors contributed to the article and approved the submitted version.

## Funding

This research was supported by funding from the German Science Foundation (DFG) Research Grants FL1051/1-1 and KA2846/6-1, as well as a Consolidator Grant of the European Research Council (ERC CoG 819585 “SYMBeetle” to MK).

## Conflict of Interest

The authors declare that the research was conducted in the absence of any commercial or financial relationships that could be construed as a potential conflict of interest.

## Publisher’s Note

All claims expressed in this article are solely those of the authors and do not necessarily represent those of their affiliated organizations, or those of the publisher, the editors and the reviewers. Any product that may be evaluated in this article, or claim that may be made by its manufacturer, is not guaranteed or endorsed by the publisher.
