# Supplementary material for: Transmission of Bacterial Symbionts With and Without Genome Erosion Between a Beetle Host and the Plant Environment
Source: Front Microbiol. 2021 Sep 22;12:715601. doi: 10.3389/fmicb.2021.715601 (PMC8493222; doi:10.3389/fmicb.2021.715601)
Supplement: Supplementary file 1 [file Data_Sheet_1.docx]

Supplementary Material

# Supplementary Data

**Supplementary Data 1.** Excel file showing the read count per sample in each beetle – plant pair for the 30 most abundant ASVs in beetle glands. These correspond to the top 30 ASVs when considering the sum of reads across beetle samples, ordered from highest to lowest abundance.

**Supplementary Data 2.** Excel file listing Ct values, amount of cDNA and calculated gene copy numbers for *Burkholderia gladioli* quantification via qPCR in sections 2.2, 2.3 and 2.4.

# Supplementary Figures and Tables

## Supplementary Figures

**
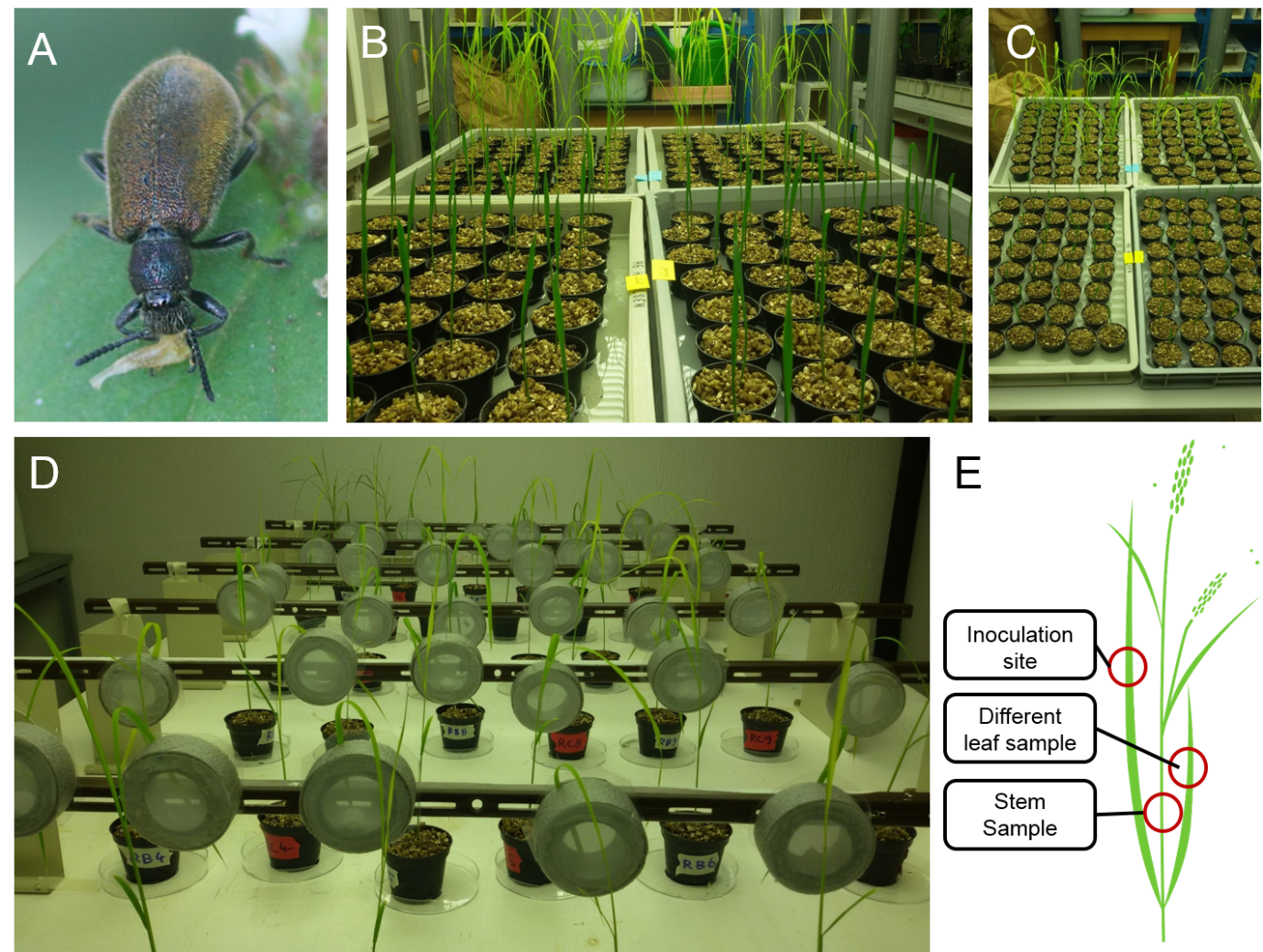
**

**Supplementary Figure 1.** (A) Adult *Lagria villosa* beetle. Picture by R. Janke. (B), (C) Rearing of wheat (front) and rice plants (back) under greenhouse conditions. Wheat plants are one week old, rice plants are three weeks old. (D) Rice plants organized in staggered order according to treatment with controlled light conditions. Clip cages were attached to metal bars with magnets. (E) Scheme of the three sampling locations, i.e. the original inoculation location (ori), the next new leaf (new) and the stem (stem).


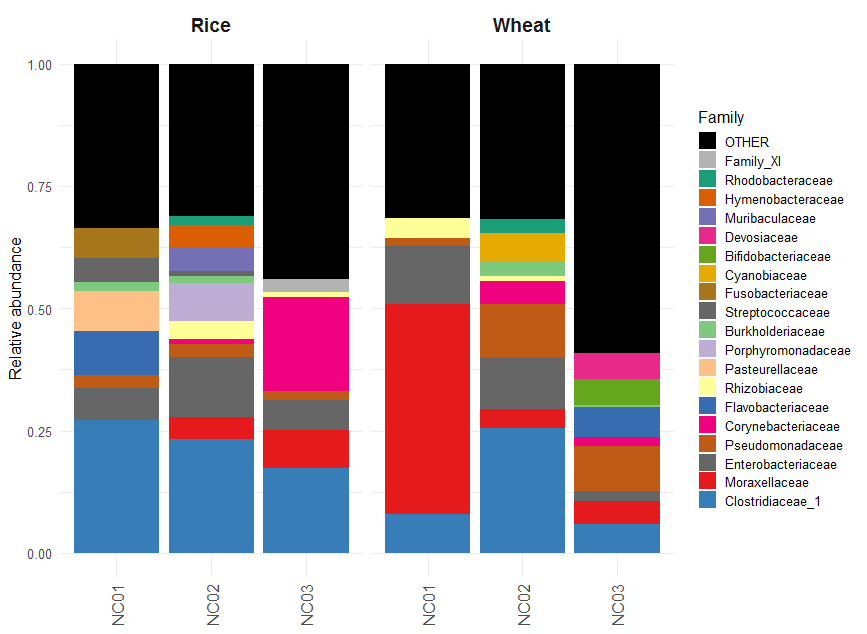


**Supplementary Figure 2.** Relative abundance of bacterial families present in rice and wheat control plants that were not exposed to *L. villosa* beetles, based on 16S rRNA high-throughput amplicon sequencing of cDNA. Only genera referred to the 30 most abundant bacterial ASVs are shown. Remaining ASVs are grouped as “OTHER”.


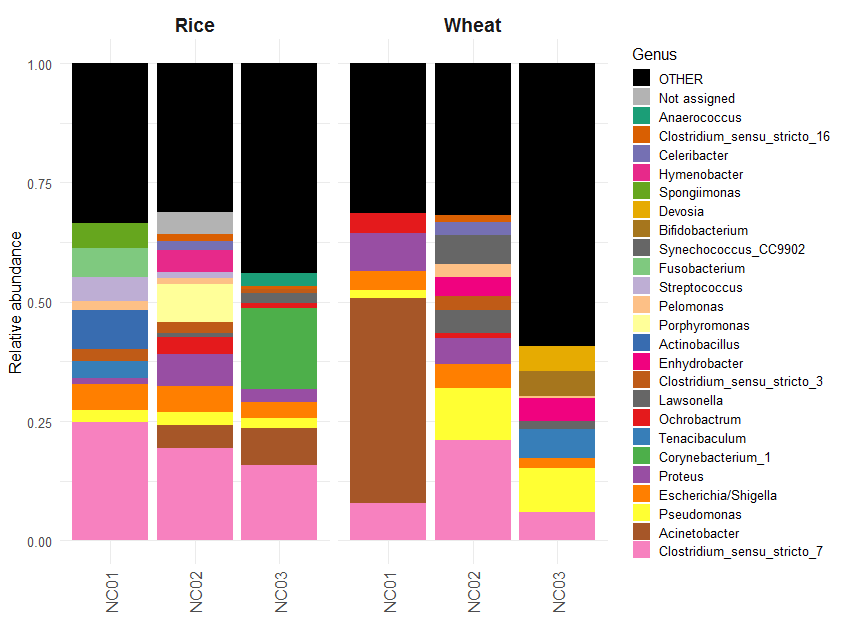


**Supplementary Figure 3.** Relative abundance of bacterial genera present in rice and wheat control plants that were not exposed to *L. villosa* beetles, based on 16S rRNA high-throughput amplicon sequencing of cDNA. Only genera referred to the 30 most abundant bacterial ASVs are shown. Remaining ASVs are grouped as “OTHER”.


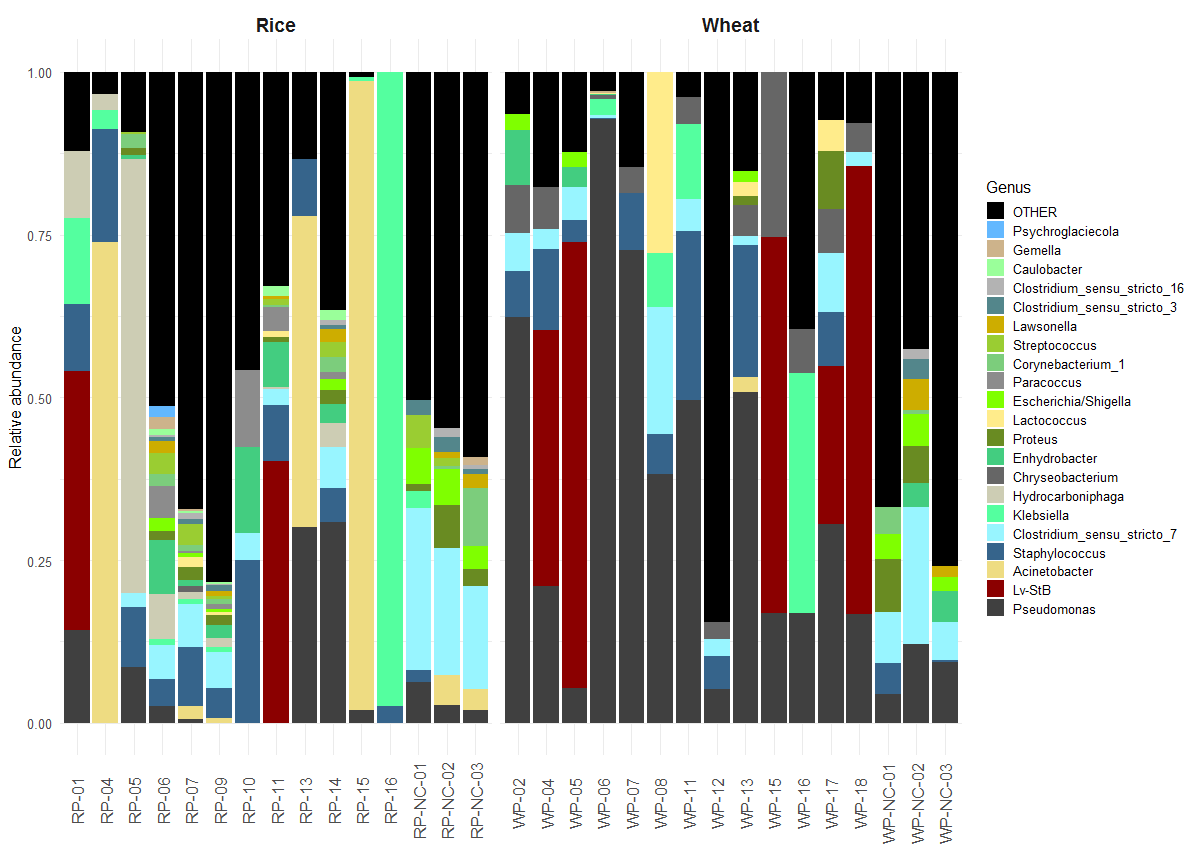


**Supplementary Figure 4.** Relative abundance of bacterial ASVs present in rice and wheat plants that were exposed to *L. villosa* beetles, identified to the genus level based on 16S rRNA high-throughput amplicon sequencing of cDNA. The 30 most abundant bacterial ASVs in plant samples are shown individually and remaining ASVs are grouped as “OTHER”.

**
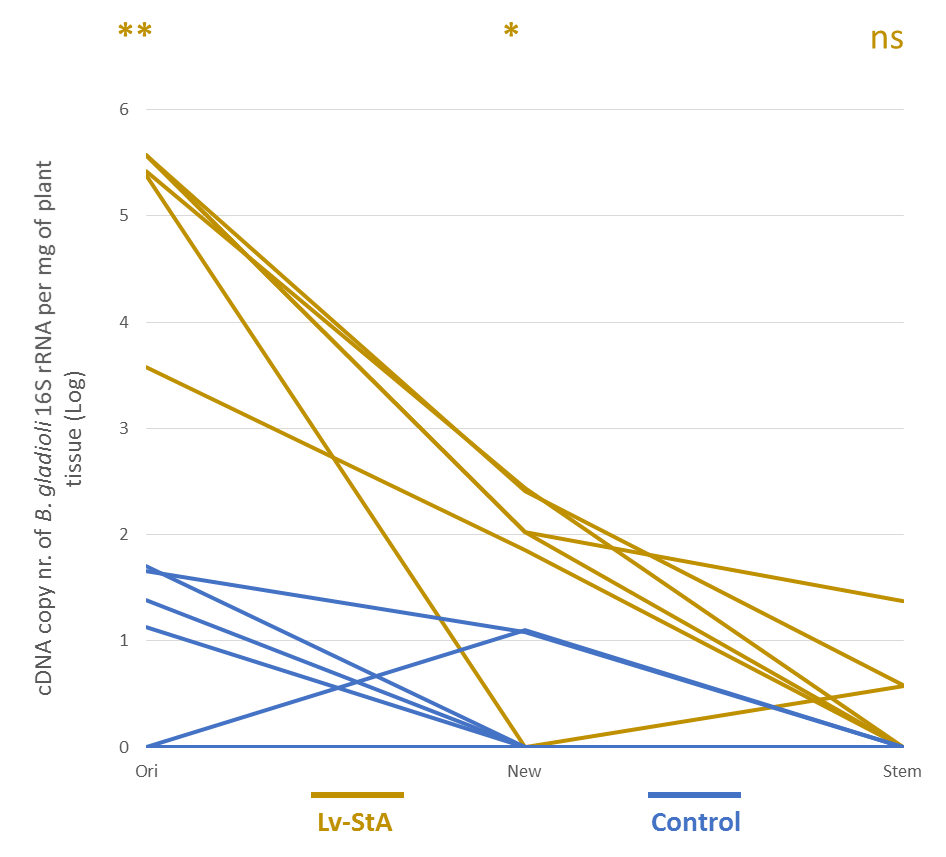
**

**Supplementary Figure 5.** Abundance of *Burkholderia gladioli* Lv-StA per mg leaf tissue in the different sample locations in wheat. The titers correspond to 16S rRNA gene copy numbers found via qPCR on cDNA, then log transformed. *B. gladioli* bacteria were found after 21 days in significantly higher amounts in the original inoculation location (Ori: Dunn’s z = 2.87; p = 0.0041) compared to uninfected controls. The same is true for the abundance of *B. gladioli* bacteria in a new leaf (New: Dunn’s z = 2.34; p = 0.0019). Also, the original leaf harbored more bacteria than the new leaf (Wilcoxon Z = -2.201, p = 0.028) and more than the stem (Wilcoxon Z = -2.201, p = 0.028).

**
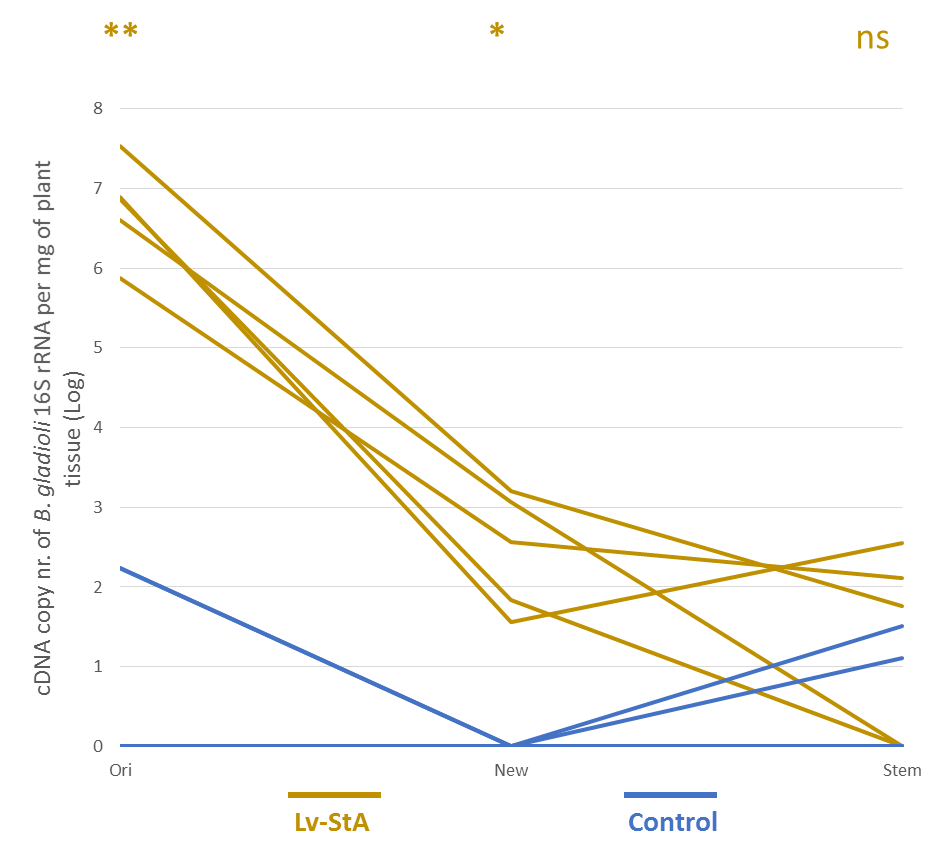
**

**Supplementary Figure 6.** Abundance of *Burkholderia gladioli* Lv-StA per mg leaf tissue in the different sample locations in rice. The titers correspond to 16S rRNA gene copy numbers found via qPCR on cDNA, then log transformed. *B. gladioli* bacteria were found after 28 days in significantly higher amounts in the original inoculation location (Ori: Dunn’s z = 3.06; p = 0.0022) compared to uninfected controls. The same is true for the abundance of *B. gladioli* bacteria in a new leaf (New: Dunn’s z = 2.83; p = 0.0045). Also, the original leaf harbored more bacteria than the new leaf (Wilcoxon Z = -2.023, p = 0.043) and more than the stem (Wilcoxon Z = -2.023, p = 0.043).

***

**

Supplementary Figure 7. Lv-StB presence in first generation lab larvae in a semi-natural environment and under controlled conditions. Offspring of field collected adults that were kept in semi-natural conditions are more likely to have Lv-StB symbionts than offspring of adults kept in plastic boxes (Terrarium 1: χ2 = 9.8462, df = 1, N_Terrarium_ = 13, N_Control_ = 13, p < 0.005; Terrarium 2: χ2 = 7.9922, df = 1, N_Terrarium_ = 24, N_Control_ = 19, p < 0.005). Dark green indicates presence, light green indicates absence of symbionts.

## Supplementary Tables

Supplementary Table 1: Thermocycler programs used.

| Name | Initial denaturation | Denaturation | Primer annealing | Elongation | Cycles | Final elongation |
| --- | --- | --- | --- | --- | --- | --- |
| Sanger | 1min 96°C | 30sec 96°C | 15sec 45°C | 4min 60°C | 25 | - |
| MKMS 50 | 3min 94°C | 30sec 94°C | 60sec 55°C | 60sec 72°C | 32 | 4min 72°C |
| MKMS 60 | 3min 94°C | 30sec 94°C | 60sec 60°C | 60sec 72°C | 32 | 4min 72°C |
| Burk3_32cy_61 | 3min 94°C | 40sec 94°C | 60sec 61°C | 60sec 72°C | 32 | 4min 72°C |
| Burk3_32cy_62 | 3min 94°C | 40sec 94°C | 60sec 62°C | 60sec 72°C | 32 | 4min 72°C |
| Burk3_50cy_62 | 3min 94°C | 40sec 94°C | 60sec 62°C | 60sec 72°C | 50 | 4min 72°C |
| qPCR Ex01 | 12min 95°C | 15sec 95°C | 20sec 65°C | 20sec 72°C | 40 | - |
| qPCR Ex02 | 12min 95°C | 15sec 95°C | 20sec 68°C | 20sec 72°C | 50 | - |

Supplementary Table 2. Specific primers used for diagnostic and quantitative PCRs.

| Primer Name | Sequence (5’-3’) | Target | use | Reference |
| --- | --- | --- | --- | --- |
| Burk16S_StA-G_F | CTGAGGGCTAAT  ATCCTTCGGGG | Lv-StA / Lh-StG 16S rRNA gene | Lv-StA and Lh-StG presence & identification | This study |
| Burk3.1Rev | TRCCATACTC  TAGCTTGC | *Burkholderia*  16S rRNA gene | *B. gladioli* presence | modified from (Salles et al. 2002) |
| Burk16S_StB_F | TTGAAGGCTAAT  ATCCTTCAAGA | Lv-StB 16S rRNA gene | Lv-StB presence & identification | This study |
| Burk16S_1_F | GTTGGCCGAT  GGCTGATT | *Burkholderia*  16S rRNA gene | *B. gladioli* presence | (Flórez et al. 2017) |
| Burk16S_1_R | AAGTGCTTTAC  AACCCGAAGG | *Burkholderia*  16S rRNA gene | *B. gladioli* presence | (Flórez et al. 2017) |
| 515F | GTGYCAGCMG  CCGCGGTAA | V4 region of eubacteria 16S rRNA gene | Identification of general bacteria | Parada et al 2016 (modified from Caporaso et al 2011) |
| 806R | GGACTACNVG  GGTWTCTAAT | V4 region of eubacteria 16S rRNA gene | Identification of general bacteria | Parada et al 2016 (modified from Caporaso et al 2011) |
| LgaG_3_fwd | CGCCGTATCG  AGCAGTTTC | Lv-StB trans-AT PKS LgaG | Lv-StB presence & identification | This study |
| LgaG_4_rev | CCTGGCGATA  ATGTGCGTAT | Lv-StB trans-AT PKS LgaG | Lv-StB presence & identification | This study |
| gla-Fw | CTGCGCCTG  GTGGTGAAG | *B. gladioli* gyrase subunit B gene | Lv-StB presence & identification | (Maeda et al. 2006) |
| gyrB-Burk_R | YTCGTTGWARCT  GTCGTTCCACTGC | *Burkholderia* gyrase subunit B gene | Lv-StB presence & identification | (Spilker et al. 2009) |
| BurkA_gyrB_F | TACTCGACCGT  GCCGAAGATA | *B. gladioli* Lv-StA gyrase subunit B gene | Lv-StA presence & identification | This study |
| BurkA_gyrB_R | GACCGCGTG  CTGGAAACG | *B. gladioli* Lv-StA gyrase subunit B gene | Lv-StA presence & identification | This study |
| C_MATK_F_1 | TAATTTACRATCAAT  TCATTCAATATTTCC | maturaseK gene in angiosperm | Plant species identification | (Heckenhauer et al. 2016) |
| C_MATK_F_4 | TAATTTMCRATCAAT  TCATTCCATATTTCC | maturaseK gene in angiosperm | Plant species identification | (Heckenhauer et al. 2016) |
| C_MATK_R_1 | GARGAYCCRCTRTRAT  AATGAGAAAGATTT | maturaseK gene in angiosperm | Plant species identification | (Heckenhauer et al. 2016) |
| rbcLa-F | ATGTCACCACAAAC  AGAGACTAAAGC | ribulose-1, 5-bisphosphate carboxylase (rbcL) gene | Plant species identification | (Levin et al. 2003) |
| rbcLa-R | GTAAAATCAA  GTCCACCRCG | ribulose-1, 5-bisphosphate carboxylase (rbcL) gene | Plant species identification | (Kress et al. 2009) |
| M13 forward | TGTAAAACGA  CGGCCAGT | pSC-A | StrataClon Kit | StrataClone |
| M13 reverse | GGAAACAGCT  ATGACCATG | pSC-A | StrataClon Kit | StrataClone |

Supplementary Table 3. Probes used for Fluorescence in situ hybridization (FISH).

| Primer Name | Sequence (5’-3’) | Target | use | Reference |
| --- | --- | --- | --- | --- |
| Burk_16S-Cy3 | TGCGGTTAGA  CTAGCCACT | *Burkholderia* 16S rRNA  5’-mod Cy3 | Localization and identification of *Burkholderia* symbionts | (Opelt et al. 2007) |
| EUB338-Cy5 | GCTGCCTCC  CGTAGGAGT | Eubacteria 16S rRNA  5’-mod Cy5 | Localization of general bacteria | modified from Amann et al. 1990 |
| Burk16S_StA-G_Cy3 | CCCCGAAGGATA  TTAGCCCTCAG | *B. gladioli* Lv-StA 16S rRNA 5’-mod Cy3 | Localization and identification of Lv-StA in leaf tissue | This study |
| Burk16S_StA_Cy5 | GCACCCTCAGAT  CTCTCCAAGG | *B. gladioli* Lv-StA 16S rRNA 5’-mod Cy5 | Localization and identification of Lv-StA in leaf tissue | This study |

Supplementary Table 4. Illumina 16S rRNA gene amplicon sequencing read count per sample. Read numbers after each filtering step are reported. The top 30 ASVs correspond to those most abundant in beetle glands considering total number of reads across all samples.

| **Sample** | **Raw** | **Quality filtering** | **Unspread** | **Chloroplast and mitochondria filtering** | **Top30-ASVs** |
| --- | --- | --- | --- | --- | --- |
| **Wheat_beetle_01** | 81546 | 64480 | 64327 | 64298 | 63537 |
| **Wheat_beetle_02** | 87220 | 71584 | 71499 | 71499 | 69001 |
| **Wheat_beetle_04** | 70624 | 55148 | 55031 | 55031 | 54388 |
| **Wheat_beetle_05** | 67793 | 56358 | 56255 | 56255 | 56157 |
| **Wheat_beetle_06** | 105909 | 83521 | 83435 | 83428 | 83323 |
| **Wheat_beetle_07** | 174068 | 153209 | 153155 | 152954 | 151346 |
| **Wheat_beetle_08** | 91441 | 74678 | 74598 | 74589 | 74564 |
| **Wheat_beetle_09** | 63965 | 50340 | 50243 | 50243 | 50237 |
| **Wheat_beetle_10** | 94574 | 74930 | 74738 | 73997 | 73570 |
| **Wheat_beetle_11** | 88693 | 70685 | 70564 | 70559 | 69113 |
| **Wheat_beetle_12** | 75634 | 62722 | 62614 | 62614 | 62541 |
| **Wheat_beetle_13** | 123139 | 102636 | 102544 | 102544 | 102423 |
| **Wheat_beetle_14** | 93375 | 74260 | 74127 | 74127 | 74043 |
| **Wheat_beetle_15** | 98769 | 80730 | 80595 | 80586 | 80571 |
| **Wheat_beetle_16** | 55442 | 44517 | 44372 | 44372 | 44343 |
| **Wheat_beetle_17** | 57317 | 48433 | 48298 | 48298 | 48298 |
| **Wheat_beetle_18** | 105577 | 84638 | 84534 | 84534 | 84478 |
| **Wheat_beetle_Extraction_ctrl_01** | 1284 | 991 | 904 | 904 | 330 |
| **Wheat_beetle_Extraction_ctrl_02** | 4738 | 3667 | 3564 | 3564 | 264 |
| **Wheat_plant_01** | 87545 | 76746 | 76668 | 6 | 6 |
| **Wheat_plant_02** | 65603 | 58678 | 58583 | 356 | 203 |
| **Wheat_plant_04** | 71088 | 62771 | 62647 | 525 | 350 |
| **Wheat_plant_05** | 68412 | 58255 | 58120 | 643 | 538 |
| **Wheat_plant_06** | 70262 | 59703 | 59585 | 6851 | 5406 |
| **Wheat_plant_07** | 61139 | 52744 | 52631 | 595 | 327 |
| **Wheat_plant_08** | 77069 | 68487 | 68380 | 97 | 91 |
| **Wheat_plant_09** | 87461 | 78646 | 78529 | 0 | 0 |
| **Wheat_plant_10** | 73754 | 64448 | 64320 | 8 | 0 |
| **Wheat_plant_11** | 62095 | 54398 | 54263 | 311 | 205 |
| **Wheat_plant_12** | 63578 | 54879 | 54756 | 418 | 33 |
| **Wheat_plant_13** | 60251 | 53230 | 53091 | 465 | 283 |
| **Wheat_plant_14** | 98045 | 85695 | 85572 | 0 | 0 |
| **Wheat_plant_15** | 90478 | 82189 | 82075 | 71 | 53 |
| **Wheat_plant_16** | 88893 | 78921 | 78814 | 268 | 144 |
| **Wheat_plant_17** | 67490 | 58438 | 58286 | 190 | 130 |
| **Wheat_plant_18** | 77593 | 66683 | 66525 | 227 | 199 |
| **Wheat_plant_Unexposed_ctrl_01** | 71895 | 60568 | 60434 | 470 | 68 |
| **Wheat_plant_Unexposed_ctrl_02** | 46488 | 41141 | 41000 | 2907 | 790 |
| **Wheat_plant_Unexposed_ctrl_03** | 54156 | 48146 | 48026 | 2579 | 54 |
| **Rice_beetle_01** | 69046 | 55460 | 55341 | 55341 | 55264 |
| **Rice_beetle_03** | 109192 | 89876 | 89741 | 89741 | 78739 |
| **Rice_beetle_05** | 80631 | 64452 | 64315 | 64309 | 63158 |
| **Rice_beetle_06** | 60135 | 50226 | 50128 | 50128 | 50128 |
| **Rice_beetle_07** | 36757 | 29259 | 29087 | 29087 | 29087 |
| **Rice_beetle_09** | 56715 | 47364 | 47282 | 47282 | 47093 |
| **Rice_beetle_10** | 51848 | 42458 | 42345 | 42338 | 42338 |
| **Rice_beetle_11** | 77477 | 61430 | 61314 | 61314 | 61314 |
| **Rice_beetle_12** | 103874 | 81472 | 81355 | 81355 | 81232 |
| **Rice_beetle_13** | 128119 | 101887 | 101758 | 101749 | 99986 |
| **Rice_beetle_14** | 125193 | 102402 | 102237 | 102095 | 101711 |
| **Rice_beetle_15** | 115701 | 93741 | 93632 | 93569 | 93446 |
| **Rice_beetle_16** | 143323 | 113959 | 113912 | 113881 | 113476 |
| **Rice_beetle_Extraction_ctrl_01** | 2257 | 1640 | 1612 | 1612 | 770 |
| **Rice_beetle_Extraction_ctrl_02** | 6546 | 5504 | 5504 | 4298 | 101 |
| **Rice_beetle_Extraction_ctrl_03** | 5361 | 4856 | 4856 | 4520 | 478 |
| **Rice_plant_01** | 73408 | 62852 | 62751 | 98 | 62 |
| **Rice_plant_03** | 73718 | 65245 | 65170 | 7 | 0 |
| **Rice_plant_04** | 61467 | 54020 | 53933 | 576 | 456 |
| **Rice_plant_05** | 75059 | 62040 | 61956 | 2530 | 1742 |
| **Rice_plant_06** | 44332 | 37906 | 37800 | 18779 | 3060 |
| **Rice_plant_07** | 90295 | 69338 | 69247 | 13972 | 1587 |
| **Rice_plant_09** | 67843 | 54571 | 54485 | 3658 | 360 |
| **Rice_plant_10** | 74092 | 62644 | 62549 | 144 | 39 |
| **Rice_plant_11** | 69642 | 58597 | 58425 | 4535 | 2203 |
| **Rice_plant_12** | 74816 | 64814 | 64738 | 5 | 0 |
| **Rice_plant_13** | 79679 | 72875 | 72798 | 90 | 70 |
| **Rice_plant_14** | 90156 | 76403 | 76311 | 2321 | 295 |
| **Rice_plant_15** | 64495 | 54873 | 54785 | 2885 | 2862 |
| **Rice_plant_16** | 84771 | 75787 | 75723 | 319 | 311 |
| **Rice_plant_Unexposed_ctrl_01** | 67807 | 60161 | 60083 | 3313 | 940 |
| **Rice_plant_Unexposed_ctrl_02** | 52667 | 47669 | 47598 | 3341 | 767 |
| **Rice_plant_Unexposed_ctrl_03** | 90285 | 78297 | 78220 | 6249 | 1127 |
